# Emotional intelligence and English as a second/foreign language learning: a systematic review using TCCM framework

**DOI:** 10.3389/fpsyg.2025.1722555

**Published:** 2026-01-09

**Authors:** G. Deepika, J. Mary Jennifer

**Affiliations:** Department of English, School of Social Sciences and Languages, Vellore Institute of Technology, Vellore, India

**Keywords:** emotional intelligence, EI, EFL, ESL, English language learning, second language learning, foreign language learning, TCCM framework

## Abstract

Emotional intelligence (EI) has been widely studied in the context of English as a second/foreign language (ESL/EFL) learning when the focus shifts from unidimensional cognitive aspects to multiple aspects, including the role of affective factors in language learning. This systematic review synthesizes 121 research articles related to EI and ESL/EFL learning and analyses the articles using the theory, context, characteristics, and methodology (TCCM) framework. The theory section outlines the theoretical frameworks integrated in the studies. The context highlights the contributions of various countries to the field and examines the study population. The characteristics section analyses the different variables of ESL/EFL investigated with EI, and the methodology addresses the data collection method, methods used to measure EI, and data analysis techniques. From the 121 selected articles, it is evident that the integration of theoretical frameworks began in the last decade. The broaden-and-build theory of positive emotions and control-value theory have been widely employed to understand how affective factors influence SLL/FLL. The majority of the studies were conducted in Iran (69), where English is a foreign language. Limited studies (six) have been conducted among school students, and all these studies were conducted among high school and secondary school students above the age of 12. Although EI has been examined in relation to various cognitive, affective, and environmental factors associated with ESL/EFL, it has been widely investigated in relation to oral communication skills and second/foreign language anxiety (SLA/FLA). The survey method (94) has been extensively used to collect data, whereas correlation (77) is a widely employed statistical test. The selected articles other than intervention studies can be categorized into four types based on their characteristics: (1) EI investigated in relation to other ESL/EFL variables; (2) EI examined with other factors, such as motivation and self-esteem, in relation to ESL/EFL variables; (3) EI explored as a mediating variable between the two constructs; and (4) studies that investigate the interrelationship among EI and other ESL/EFL variables. This review also discusses future research agendas pertaining to each construct in the TCCM framework.

## 1 Introduction

Language learning, particularly a foreign or second language, is a complex cognitive process (Yu, 2022) in which learners encounter both positive (amusement, awe, enjoyment/joy, empathy, gratitude, hope, inspiration, interest, love, mindfulness, pride, serenity, and wellbeing) and negative emotions (anxiety, anger, burnout, boredom, stress, contempt, disgust, embarrassment, feeling scared, fear, guilt, hate, sadness, shame, and tension) (MacIntyre and Vincze, 2017). Wang et al. (2024) stated that emotions play a vital role in all aspects of second/foreign language learning (SLL/FLL), which resulted in multitude of studies on the impact of affective factors in SLL/FLL. Dewaele and Li (2020) categorized emotion research in second language acquisition into three phases: (i) avoidance; (ii) anxiety-prevailing; and (iii) positive/negative emotions. In the first phase, studies neglected the role of emotions, focusing on cognitive aspects. In the second phase, anxiety attracted researchers (Yu, 2022), and numerous studies on effective ways to mitigate SLA/FLA, the causes of SLA/FLA, and how alleviating anxiety influences SLL/FLL were conducted (Cheng et al., 1999; Horwitz, 2001; MacIntyre and Gardner, 1991, 1994; Woodrow, 2006). In the third phase, importance has been given to both negative and positive emotions. The widespread acceptance of positive psychology in the late twentieth and early twenty-first centuries (Linley et al., 2006) emphasizes the importance of positive emotions in second language learning, and it is evident that the focus of SLL/FLL researchers has shifted from negative emotions to negative and positive emotions, particularly anxiety and enjoyment (Li, 2019; Resnik and Dewaele, 2020; Li et al., 2021; Resnik and Dewaele, 2021; Wang and Wang, 2024).

Activities which learners enjoy and perform with interest positively influence language learning outcomes (Song, 2024; Wang et al., 2023). A longitudinal study (Tsang and Lo, 2025) among Hong Kong primary learners showed that learner-initiated informal exposure to English through videos and reading is significantly associated with vocabulary gains in 1 year, especially at the two-thousand-word level, whereas time spent in tutorials and doing homework does not show positive associations with total vocabulary gains. Considering the socioeconomic context, the study also found that learners from households with higher incomes tended to have more informal exposure; however, parental education did not influence exposure levels. Chan and Lo (2024) highlight the role of digital motivation design as an environmental pathway shaping learners' emotions and engagement in a systematic review of thirty SSCI indexed articles on gamification in ESL/EFL contexts from 2010 to 2022. The findings revealed that incorporating game elements such as points, badges, immediate feedback, progress bars, and leaderboards consistently increased learners' motivation and engagement and frequently reduced anxiety levels. Second language learners simultaneously experience both positive and negative emotions which interact with one another depending on their intensity (Dewaele and MacIntyre, 2014; MacIntyre and Gregersen, 2012). Understanding and regulating emotions (components of EI) are vital in SLL/FLL learning, because individuals with high EI can alleviate negative emotions and generate positive emotions (Li and Xu, 2019), which further enhances the learning process and results in better performance.

EI is defined as the ability to perceive, understand, express, and regulate one's own and others' emotions (Mayer and Salovey, 1997). EI predicts growth and development in every aspect of human life, including physical health (Keefer et al., 2009), wellbeing (Di Fabio and Kenny, 2016), job satisfaction (Suleman et al., 2020), work-life balance (Koubova and Buchko, 2013), and education (Quílez-Robres et al., 2023). The inseparable and interdependent nature of emotion and cognition (Brosch et al., 2013) has resulted in a multitude of studies on the interconnection between EI and academic performance and achievement (Ononye et al., 2022; Shengyao et al., 2024; Yee Von et al., 2022), academic stress (Khorasani et al., 2023; Roy et al., 2021; Rao, 2022; Stevens et al., 2019), and test anxiety (Abdollahi and Abu Talib, 2015; Khaledian, 2013; Shah et al., 2017; Thomas et al., 2017; Tom and Ansia, 2017; Trigueros et al., 2020). EI can potentially impact students' wellbeing, happiness, optimism, social support, self-directed learning, and interpersonal relationships (Yang and Duan, 2023). EI which comprises self-awareness, self-regulation, motivation, empathy, and social skills (Goleman, 1995), influences SLL/FLL by shaping learners' emotional experiences, social interactions, and coping strategies in the language classroom (Oxford, 2016; Pishghadam, 2009). Self-awareness and self-regulation are essential to identify one's language skills, which helps to focus on areas that need improvement (MacIntyre and Gregersen, 2012; Oxford, 2017). Studies have proven that motivation plays an important role in SLL/FLL (Dörnyei, 2005; Dörnyei and Ushioda, 2011). According to sociocultural theory, empathy and social skills have a significant impact on SLL/FLL learning (Gkonou et al., 2020; Lantolf and Thorne, 2006). Furthermore, investigations on the relationship between EI and the cognitive, affective, and environmental variables of SLL/FLL denote the importance of EI in the language learning process (Dewaele and MacIntyre, 2014; Li and Xu, 2019). Among the numerous languages, English has become the world's second language (Krashen, 2003), and individuals in non-English-speaking countries widely learn it as a second or foreign language. This study systematically reviews the existing literature on EI and English as a second/foreign language (ESL/EFL) learning using the theory, context, characteristics, and methodology (TCCM) framework.

A few review articles have examined EI in the context of SLL/FLL learning. Peng and Shuhong (2025) in the meta-analysis of EI and language achievement, by reviewing 47 independent studies, identified that there exists a significant correlation between EI and subjective language achievement, whereas the relationship is moderate between EI and objective language achievement. (Babanoğlu 2025) stated that enhancing EI among learners is important because a meta-analysis revealed a strong negative correlation between EI and FLA. Nurturing EI helps to mitigate anxiety, a key element that hinders language learning. Yu (2022) reviewed two common emotions experienced by language learners in the classroom–FLA, a negative emotion, and FLE, a positive emotion–with their influencing nature, dynamicity, and the relationship between them. In addition, the study showed that EI acts as a mediator of the multiple emotions experienced by foreign language learners. In a meta-analytic review of EI and second/foreign language achievement, Wang and Liu (2023) observed a medium to strong relationship between EI and language achievement. Wang and Wang (2022) presented a meta-analysis of the interrelationships between foreign language teachers' EI, self-efficacy, and burnout. This study found that EI was positively correlated with self-efficacy and negatively correlated with burnout. Kotsou et al. (2018) systematically reviewed 46 intervention studies on improving EI among adult population and stated that the outcome of the studies cannot be generalized as the selected studies had important limitations. Furthermore, the insights and constraints of the studies, along with suggestions for future interventions are discussed. Dewaele et al. (2008) reviewed and studied the impact of trait EI and socio-biographical variables on communicative anxiety (CA) and FLA; and identified that adults with high EI levels possess low level of CA/FLA. The review articles mentioned above focused on meta-analyses, systematic reviews of experimental studies, and thematic reviews of the impact of EI on CA, FLE, and FLA. Previous reviews have focused on synthesizing the effect size of EI on language learning variables, which clearly indicates that EI plays a significant role in language learning. However, limited attention has been paid to the conceptual and methodological landscapes. There is a gap because no review articles related to EI and ESL/EFL learning have analyzed the existing literature using the TCCM framework. Publication trends on EI related to language learning show a rapid increase in the number of publications from 2018 onwards (Peng and Shuhong, 2025). Reviewing the accumulated literature by adopting the TCCM framework is essential as it provides a structured synthesis across four dimensions: theoretical frameworks guiding the research, different educational and cultural contexts examined, characteristics of the studies, and methodologies employed. It provides a multidimensional understanding of studies related to EI and ESL/EFL learning and highlights the underexplored areas. This systematic review includes all empirical studies (quantitative descriptive, qualitative descriptive, intervention, and mixed methods) in the field of ESL/EFL learning because empirical studies generate knowledge based on evidence from systematic data collection and analysis (Creswell and Creswell, 2018; Punch, 2014). This review aims to analyse the existing literature to highlight the prevailing patterns and to identify potential areas yet to be explored by addressing gaps in theoretical frameworks, contexts, characteristics, and methods. Based on the aim, the following research questions (RQ) are framed: RQ1: what theoretical frameworks, contextual settings, and predominant study characteristics define existing research on EI and ESL/EFL learning? RQ2: what methodological approaches have been employed in studies examining EI within ESL/EFL learning contexts?

The objective of this study is to analyse studies related to EI and ESL/EFL learning using the TCCM framework to identify trends and patterns over the years. The following research objectives (RO) were formulated to address the research questions: RO1: to identify and synthesize the theoretical frameworks, contextual settings, and predominant study characteristics defining existing research on EI and ESL/EFL learning. RO2: to analyse and categorize the methodological approaches employed in studies related to EI and ESL/EFL learning. Based on the research objectives, the following review assumptions (RA) were established: RA1: diverse theoretical frameworks have been employed in the studies to understand the relationship and influence of EI in ESL/EFL learning; RA2: research on EI and ESL/EFL learning has been conducted across different countries where English is learnt as a second/foreign language and among different study populations; RA3: the impact of EI on all four language skills and the influence of EI on cognitive, affective, and environmental variables related to ESL/EFL have been widely investigated; RA4: the use of data collection methods, measurement tools, and statistical tools has evolved significantly.

## 2 Research method

### 2.1 Study design

**Figure 1:**
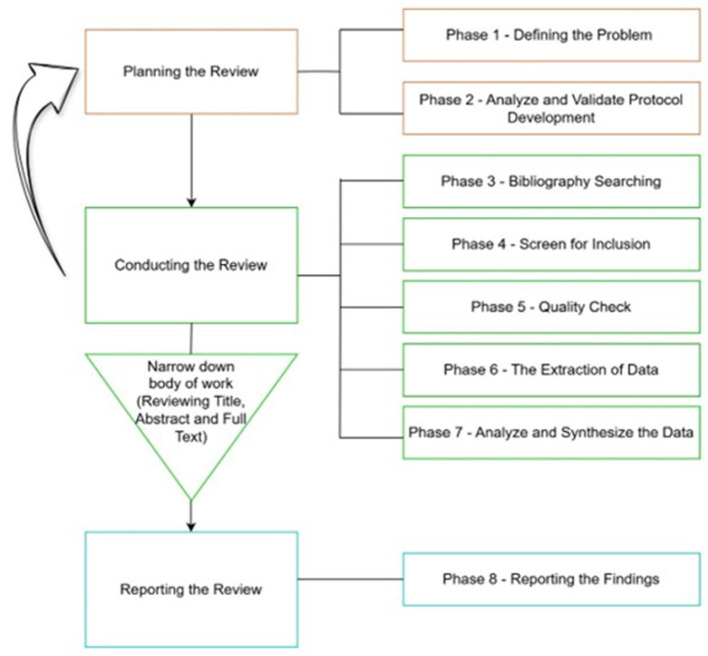
The eight-step process of conducting a literature review (Xiao and Watson, 2019).

Systematic reviews implement explicit and organized methods to collate and synthesize the findings of previous research to address a clearly stated research question. It follows strategies that minimize bias in assembling, examining, and synthesizing existing literature on specific topics (Chalmers et al., 2002). This study adopted an eight-step process (Figure 1) to conduct a systematic review (Xiao and Watson, 2019) to ensure the validity and reliability of the study. The Preferred Reporting Items for Systematic Reviews and Meta-Analyses (PRISMA) guidelines were employed to ensure transparency and trustworthiness and to pave the way for replication (Page et al., 2021). Existing literature on EI and ESL/EFL learning was identified, and studies were selected for this study by following the PRISMA guidelines.

### 2.2 Search strategy

The current review used three databases: Scopus, Web of Science, and ERIC. Two widely recognized databases, Scopus and Web of Science (Singh et al., 2021), were selected owing to their reliability, extensive content, and global citation metrics (Guimarães et al., 2025). ERIC, has also been included as it provides access to a wide range of journal articles and conference papers in the field of education. The literature search was conducted in March 2025, and to include recently published studies, it was conducted in October 2025. The keywords employed for searching the articles in the databases included: (1) “Emotional intelligence”/EI/“emotional competence”/“socio-emotional”; AND (2) “Foreign Language”/“Second Language”/“Language Learning”/“English language”/FLL/SLL/ELL/EFL/ESL/listening/speaking/L2/reading/ writing/TESOL. Search strings employed in each database are—Scopus: (TITLE-ABS-KEY (“emotional intelligence” OR “ei” OR “emotional competence” OR “socio-emotional”) AND TITLE-ABS-KEY (“second language” OR “language learning” OR “English language” OR “foreign language” OR “listening” OR “speaking” OR “reading” OR “writing” OR “ELL” OR “SLL” OR “FLL” OR “ESL” OR “EFL” OR “L2” OR “TESOL”)); ERIC: (“emotional intelligence” OR “ei” OR “emotional competence” OR “socio-emotional”) AND (“foreign language” OR “second language” OR “language learning” OR “listening” OR “reading” OR “writing” OR “speaking” OR “ESL” OR “EFL” OR “FLL” OR “ELL” OR “L2” OR “TESOL” OR “SLL”); and Web of Science: “Emotional Intelligence” OR “ei” OR “emotional competence” OR “socio-emotional” (All Fields) and “second language” OR “foreign language” OR “language learning” OR “English language” OR “listening” OR “speaking” OR “reading” OR “writing” OR “ELL” OR “ESL” OR “EFL” OR “FLL” OR “SLL” OR “L2” OR “TESOL”.

### 2.3 Screening and selection

**Figure 2:**
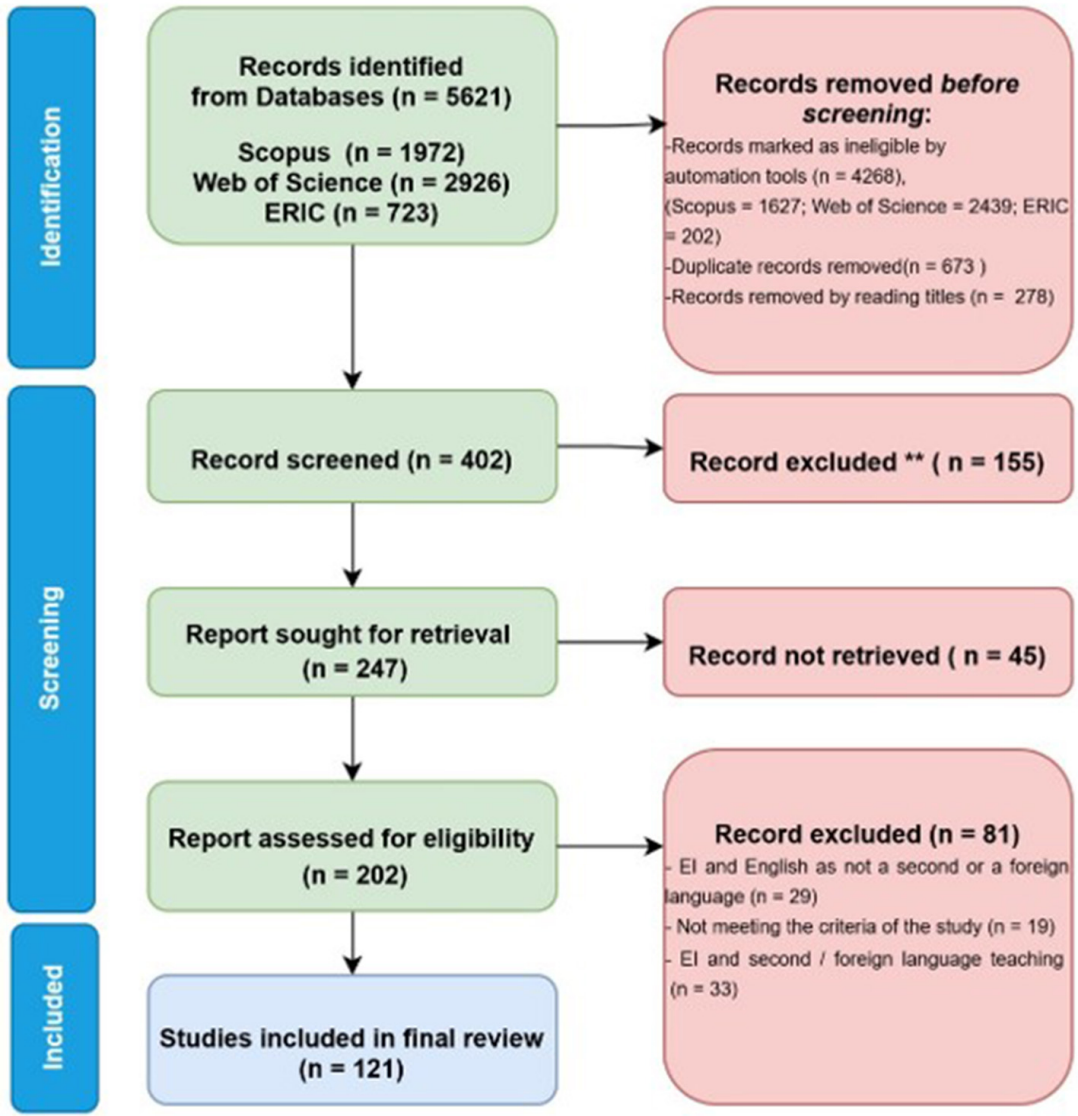
PRISMA flow diagram for systematic review from initial identification of studies from databases to final inclusion (Page et al., 2021).

A data search employing the above-listed keywords in Scopus (1,972), Web of Science–WoS (2,926), and ERIC (723) until October 2025 yielded 5,621 documents. Using automation tools, 4,268 ineligible records were excluded. Of the 1,972 documents identified in Scopus, automation tools limited the subject areas to arts and humanities, document types to articles and conference papers, and language to English, resulting in 345 documents and removing 1,627 documents. Among 2,926 documents from WoS, limiting the document types to articles or proceeding papers, limiting the WoS categories to Education Educational Research or Linguistics or Language Linguistics or Humanities Multidisciplinary, and limiting only to studies published in the English language, using automation tools finally yielded 487 documents, thereby removing 2,439 documents. In ERIC, among 723 documents resulting from the search string, selecting only peer-reviewed documents resulted in 521 documents, thereby removing 202 articles. Using automation tools in all three databases, 4,268 studies were removed. Subsequently, documents from all three databases were merged, and 673 duplicates were removed. After reading the titles, 278 studies were removed, and 402 documents were selected for abstract screening. The abstracts of 402 articles were skimmed independently, and 155 studies were excluded based on the exclusion criteria. Subsequently, 247 articles were sought for retrieval, of which 45 articles were not retrieved. A total of 202 full-text documents were reviewed. After skimming the full documents, 81 studies were excluded based on the criteria, and finally, 121 articles which met all the inclusion criteria were selected for the study. The PRISMA flow chart (Figure 2) elucidates the step-by-step process involved in selecting the articles from databases to selecting the articles for this study by clearly indicating the inclusion and exclusion criteria used.

#### 2.3.1 Inclusion and exclusion criteria

The studies were included for this systematic review if it meets the following criteria, (i) studies focussed on EI and English as a second/foreign language learning; (ii) studies that are empirical in nature–interpretation based on primary data; and (iii) studies published in English language. The reasons for exclusion are (i) studies that do not include EI as one of the components; (ii) studies that were not empirical in nature; (iii) studies that investigated EI in an academic context (academic achievement, academic performance, engagement, academic stress) but not related to language learning; (iv) studies that measured EI but investigated second/foreign languages like Spanish, German, Mandarin, and French other than English; (v) studies that measured ESL/EFL teachers and its relationship with learning outcomes; and (vi) articles that do not meet the criteria for this review—without strong methodology.

### 2.4 Risk of bias assessment

This study primarily focuses on providing an overview of the TCCM components and does not interpret the results, such as conducting meta-analysis or calculating effect size for each study. Therefore, the studies are selected for this review, if they have clearly stated research questions and explicit research methods. Using methodological appraisal tool such as the MMAT, which requires two independent reviewers, is not feasible because the screening process has been conducted by a single reviewer. This may result in a potential risk of selection bias, as no formal methodological quality assessment has been performed. Consequently, the absence of a structured appraisal process might affect the robustness of the review's findings, and the conclusions should be interpreted considering these limitations.

## 3 Data analyses and results

The TCCM framework (Paul and Rosado-Serrano, 2019) was selected for analyzing the articles to understand the theoretical framework applied, identify the context (country and study population), find the variables of ESL/EFL that have been explored with EI, and examine the methods (research method, statistical techniques, methods to measure EI) employed in the studies. Analyzing theories, contexts, characteristics, and methods of existing literature is essential for identifying gaps and justifying new studies (Creswell and Creswell, 2018). Examining the theories in the existing literature helps to understand the theoretical frameworks that adapt one or more theories related to the conceptualization of a phenomenon (Varpio et al., 2020). Reviewing the context and characteristics of the existing literature is important because identifying the context highlights underexplored populations and circumstances, whereas investigating the characteristics of the existing literature is vital as it reveals the prevailing trends and patterns of the research. Evaluating the research methods exposes various instruments employed to measure EI and different methods used for data collection analysis.

The Theory section expounds on the different theoretical frameworks adopted in these studies. The context examines the study population (school students, college/university students, or the general population) and specifies the countries in which the previous studies were conducted. The characteristics section elucidates the nature of the study and how EI has been studied with various variables related to ESL/EFL learning. The methodology expounds the data collection methods (survey/experimental/semi-structured interview/observation), tools to measure EI (different types of scales/questionnaires), and different statistical tools (correlation/regression/SEM/ANOVA/MANOVA) employed in the selected studies.

### 3.1 Reference analysis

**Figure 3:**
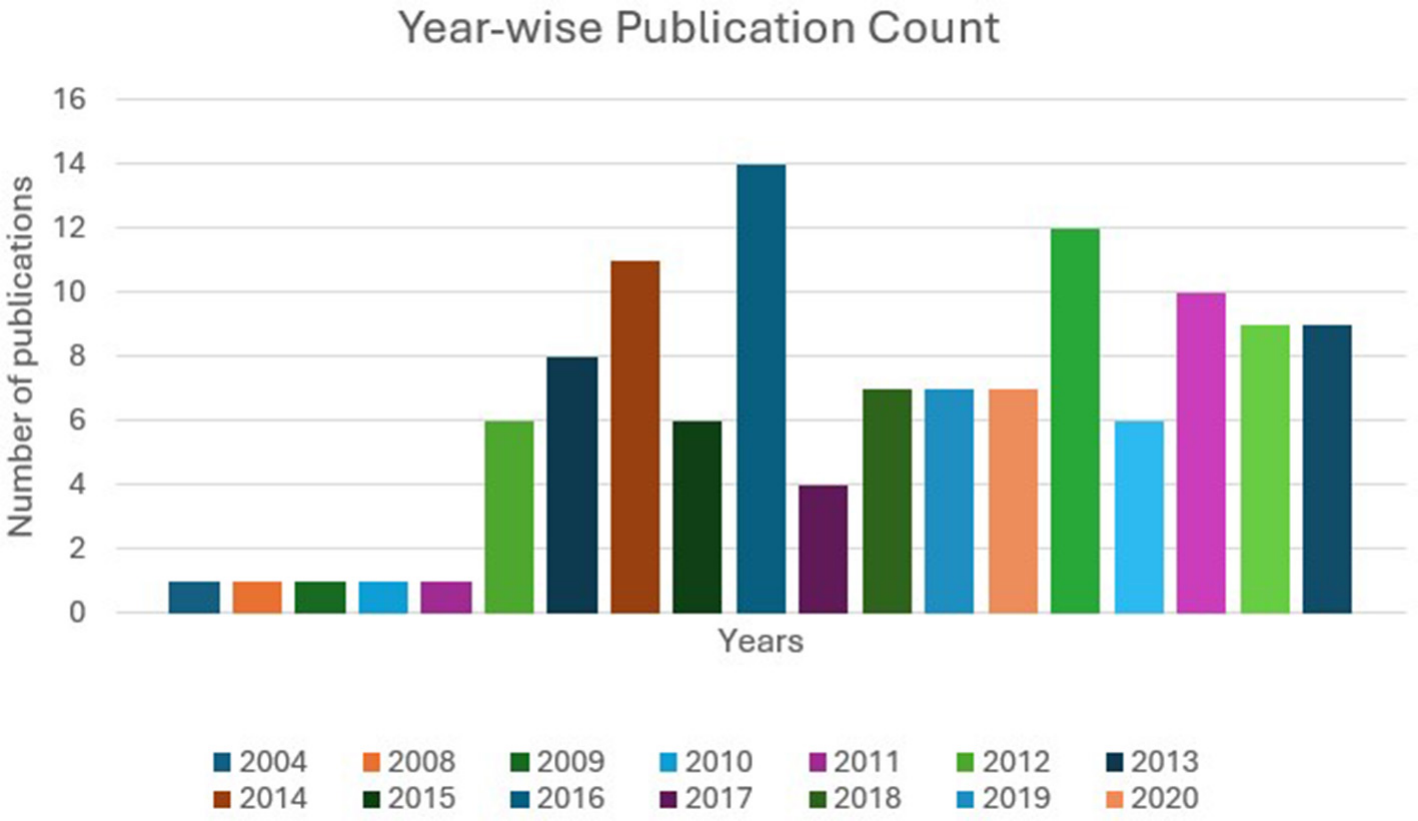
Number of publications per year from 2004 to October 2025.

Figure 3 illustrates the number of publications on EI and ESL/EFL from 2004 to October 2025. From 2004 to 2010, there were four publications; from 2011 to 2015, this number increased to 32; from 2016 to 2020, it was 39; and from 2021 to 2025 (October), it was 46. The gradual increase in the number of publications shows a growing tendency in EI and ESL/EFL learning research.

### 3.2 Theoretical framework of studies on EI and ESL/EFL

**Figure 4:**
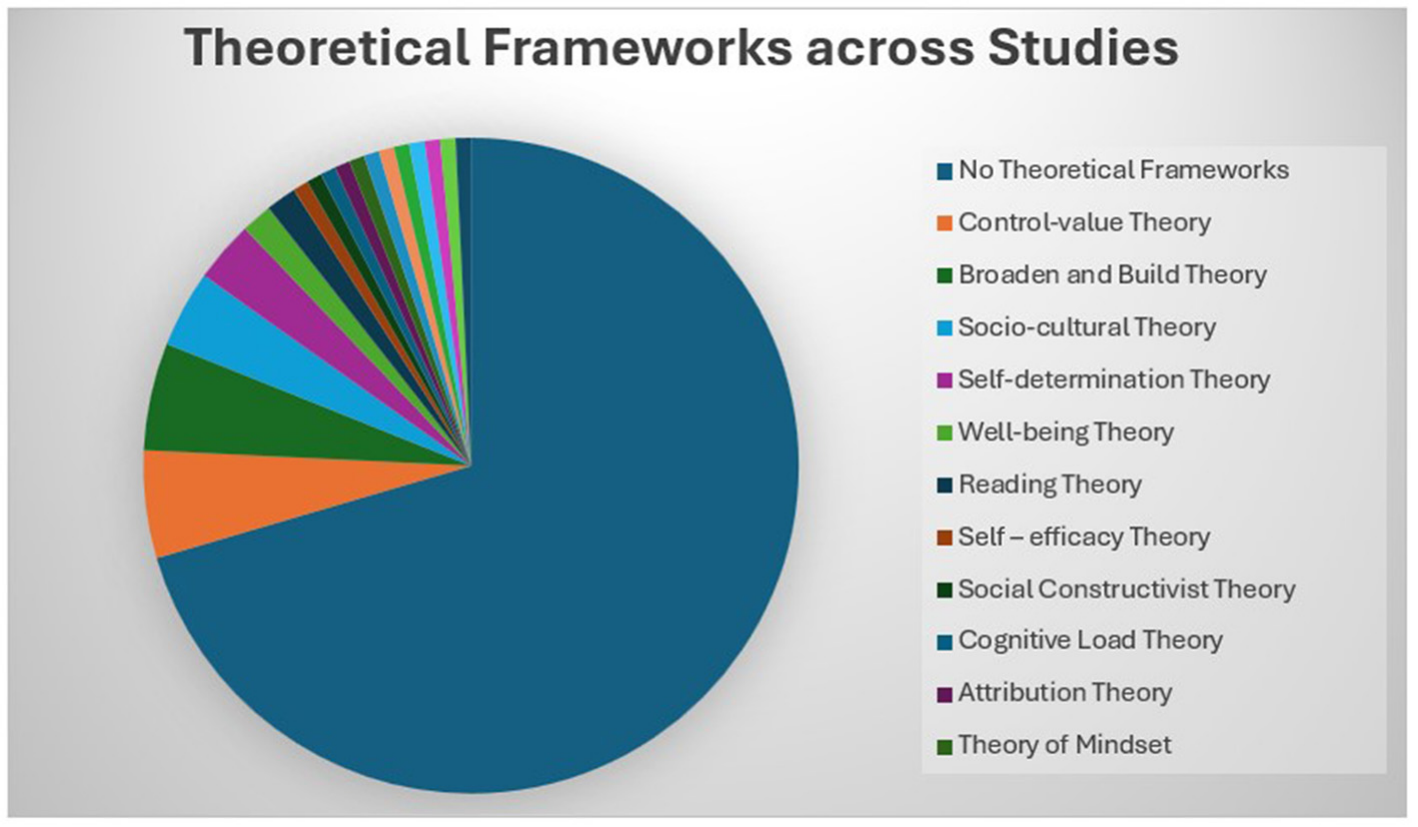
Distribution of theoretical frameworks across studies. This figure indicates that majority of studies lack theoretical frameworks.

**Figure 5:**
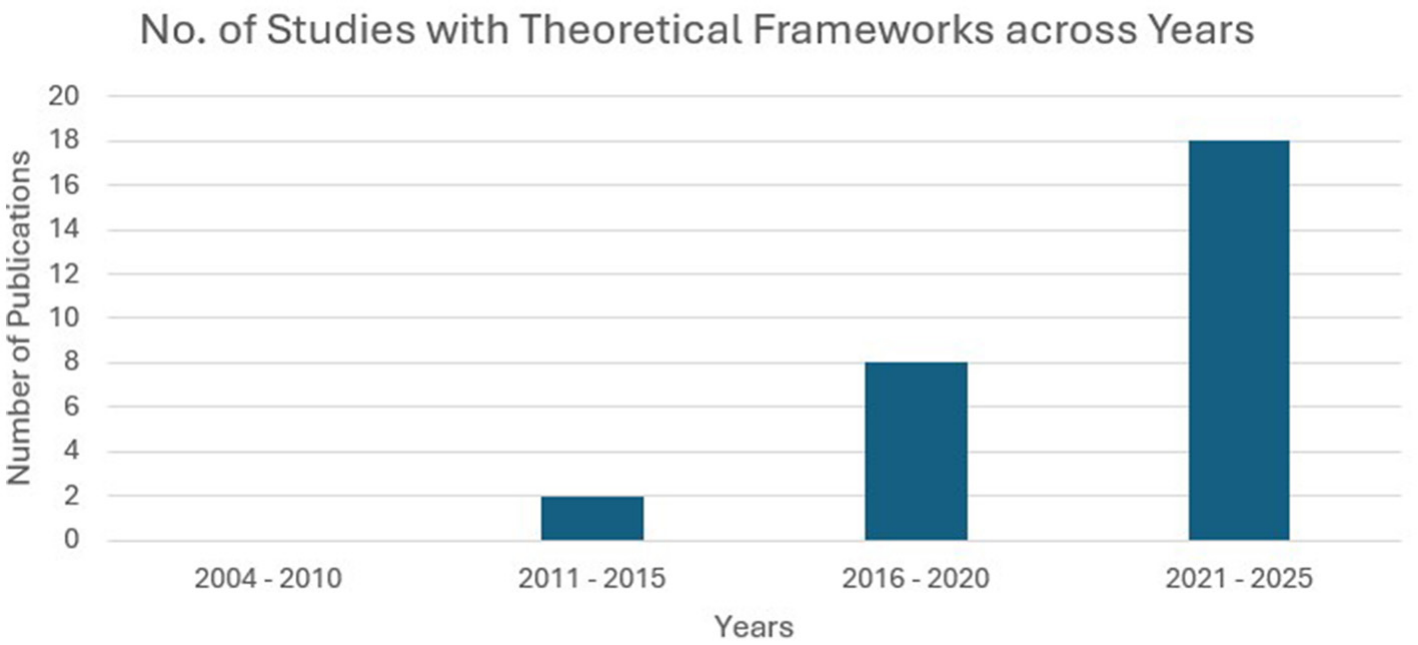
Increase in number of studies with theoretical frameworks over years. Integration of theoretical frameworks drastically increased after 2020.

A theoretical framework helps us understand the complex connections among concepts and aims to explain the abstract notion of different phenomena in various situations (Brydges and Batt, 2023). Categorizing theories in the existing literature on EI and ESL/EFL according to discipline is complicated because most of the theories employed are inherently interdisciplinary, drawing insights from psychology, education, linguistics, and the social sciences. An interdisciplinary approach to applied linguistics helps us understand complex problems and provides innovative solutions for language learning (Vijayakumar, 2024). The integration of theories from psychology and education in studies related to EI and ESL/EFL paved the way for understanding the complex relationship that exists between the two constructs. Despite the interdisciplinary nature of the field, the theories employed are generally classified as follows: psychology-based theories include control-value theory, self-efficacy theory, wellbeing theory, broaden-and-build theory, attribution theory, attachment theory, self-determination theory, theory of grand personality, and theory of mindset. Education and language learning theories include social cognitive theory, multiple intelligences theory, cognitive load theory, social constructivist theory, socio-cultural theory, socio-educational models, complex dynamic systems theory, reading theory, and schema theory. Among the 121 articles selected for this study, 23% (28) provided 18 different theoretical frameworks, of which 93% (26) were published in the last decade (2016–2025). Figure 4 shows the distribution of theoretical frameworks across selected studies and Figure 5 illustrates the evolution of integrating theoretical frameworks over years.

**Figure 6:**
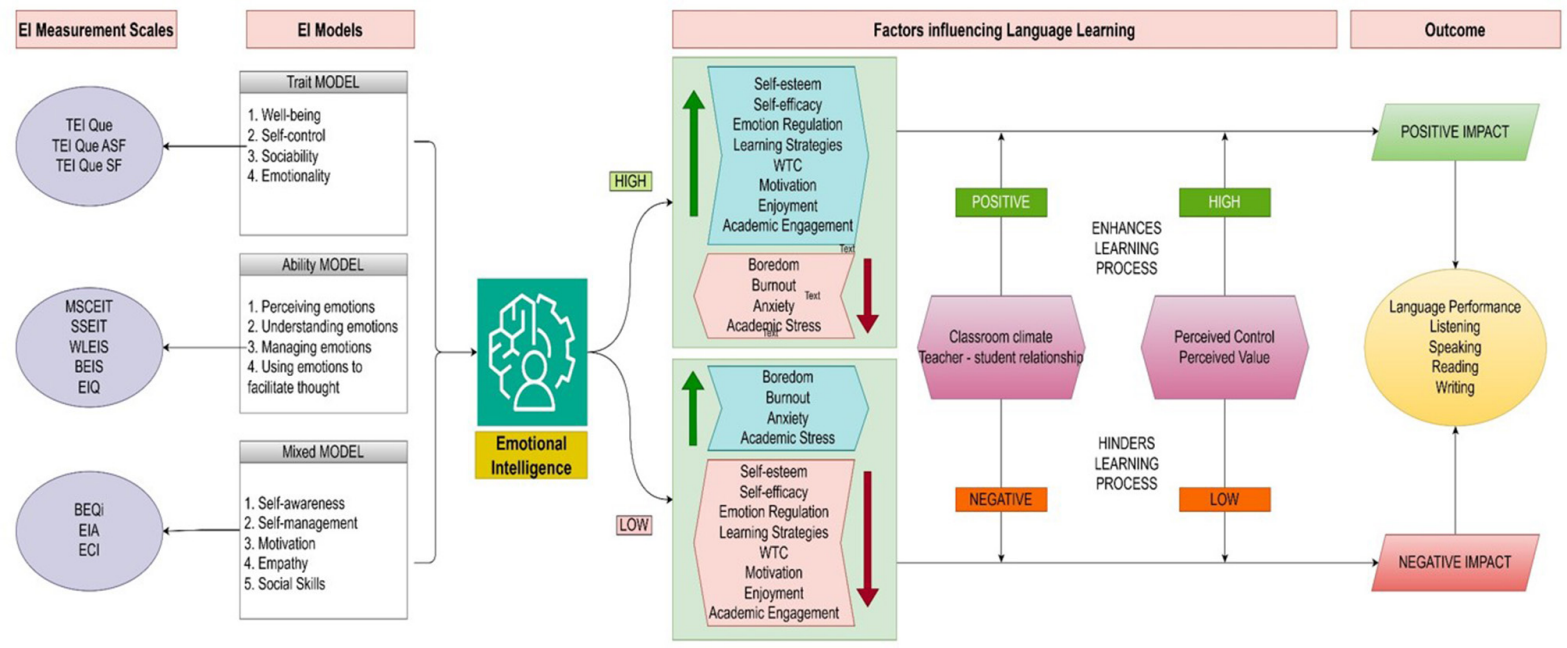
Dominant theoretical pathways tested in the studies on EI and ESL/EFL learning.

Research in this field employed theoretical frameworks in 2013 (Abdolrezapour et al., 2018; Barzegar and Sadr, 2013). Since 2019, the broaden-and-build theory of positive psychology has been widely employed in the existing literature on EI and ESL/EFL learning to study positive emotions, particularly foreign language enjoyment (FLE). A paradigm shift occurred in the last quinquennial, when positive emotions were given equal importance and studied along with negative emotions in the EFL context. The second most widely applied theory is the control-value theory which has been integrated into EI and ESL/EFL research since 2019. According to Fredrickson (2001), positive emotions: (i) broaden people's attention and thinking; (ii) undo lingering negative emotional arousal; (iii) fuel psychological resilience; (iv) build consequential personal resources; (v) trigger upward spirals toward greater wellbeing in the future; and (vi) seed human flourishing. Studies related to EI in the ESL/EFL context have found that language learners with higher EI experience a wide range of positive emotions which help counteract negative emotions, broaden attention that enhances learning, and possess good resilience, which is reflected in language achievement (Alrabai and Alamer, 2022; Han et al., 2022; Li, 2019; Li and Zhang, 2024; Resnik and Dewaele, 2021). Li and Xu (2019) examined the role of positive psychology-based activities, such as identifying three good things, developing optimism, adoring positive experiences, and the ARGUER model of nurturing EI, to investigate its impact on FLE and FLA. Pekrun (2006) stated that the emotions aroused in an academic context are based on the control and value appraisal of learners toward the subject, and self-efficacy is the predominant source of those emotions. Bandura (1977) claimed that emotion regulation, a component of EI, influences an individual's self-efficacy. Jin et al. (2024) found that learners with higher EI have better emotion regulation skills which increases their level of self-efficacy and results in improved language abilities. The broaden-and-build and control-value theories underpin the reciprocal relationship between cognitive and affective factors in SLL/FLL. Figure 6 illustrates the dominant theoretical pathways tested in the selected studies.

### 3.3 Context of studies on EI and ESL/EFL

#### 3.3.1 Countries

**Figure 7:**
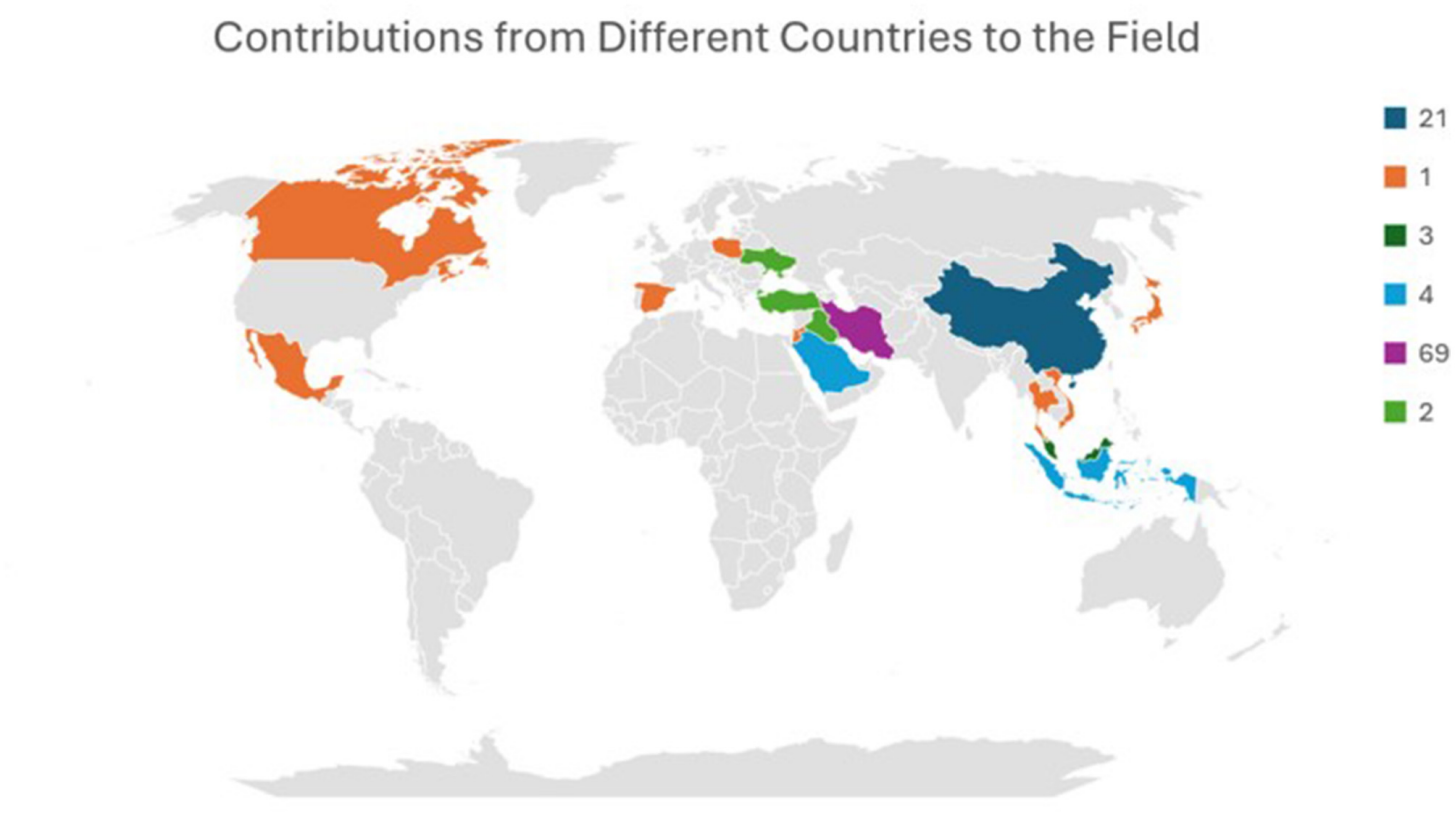
Contributions from countries across the world. Iran has significantly contributed to the domain next to China.

Research on the relationship between EI and ESL/EFL been conducted in 16 countries worldwide. The majority of the research (78) was conducted in Middle Eastern countries, including Iran, Iraq, Saudi Arabia, Jordan, and Türkiye. Asian countries, including China, Japan, and Southeast Asian countries (Thailand, Indonesia, Malaysia, and Vietnam), have explored this phenomenon in 31 research articles. Ten studies were conducted in European countries, including Ukraine, Spain, and Poland, of which six were conducted across multiple countries. Among studies from different countries in the Middle East, Asia, and Europe, only two studies were conducted in North American countries (Canada and Mexico). Figure 7 depicts the contribution of different countries to the field.

From the contextual analysis of existing studies, many studies (74%) were conducted in Iran (57%−69%) and China (17%−21%). However, among the 21 studies conducted in China, 20 were published after 2019. The recent rapid increase in the number of studies indicates the emerging and growing tendency of studies related to EI and EFL in China. The education systems in both Iran and China are traditionally teacher-centered and exam-oriented (Pishghadam and Mirzaee, 2008; Wang, 2007), which is closely associated with high anxiety and low enjoyment and creates stress in EFL classrooms (Pishghadam et al., 2013; Yan and Horwitz, 2008). Persian/Farsi is the national and official language of Iran; eventually, Iranians learn EFL in schools or private language institutes (Moharami and Daneshfar, 2021). China's Ministry of Education recommends English as a compulsory language from the third grade and expands the language by enabling it as a medium of instruction in higher education in multilingual universities (Jablonkai and Hou, 2023). In these countries, students learn English because it is vital to pursue higher education in distinguished institutions and career development (Shao et al., 2013a; Dewaele and Li, 2020). Investigating learners' EI in this context provides insight into the affective barriers that hinder language learning, and addressing these barriers facilitates the learning process. The sociocultural beliefs and educational philosophies of Iran (Islamic) and China (Confucian) emphasize empathy, social harmony, and emotion regulation, which strongly align with EI (Ghanizadeh and Moafian, 2010; Ghanizadeh and Li, 2019). Six studies were conducted in multiple countries that compared the level of EI with autonomy and emotions in online and in-person teaching of EFL (Resnik and Dewaele, 2021); self-perception in the target language (Resnik et al., 2025); CA and FLA among multilinguals (Dewaele et al., 2008); anxiety and enjoyment in L1 and L2 classes (Resnik and Dewaele, 2020); “feeling different” while communicating in a foreign language among bilinguals and multilinguals (Ożańska-Ponikwia, 2012); and EI act as a mediator between FL grit and FLA/FLE (Resnik et al., 2021).

#### 3.3.2 Population

**Figure 8:**
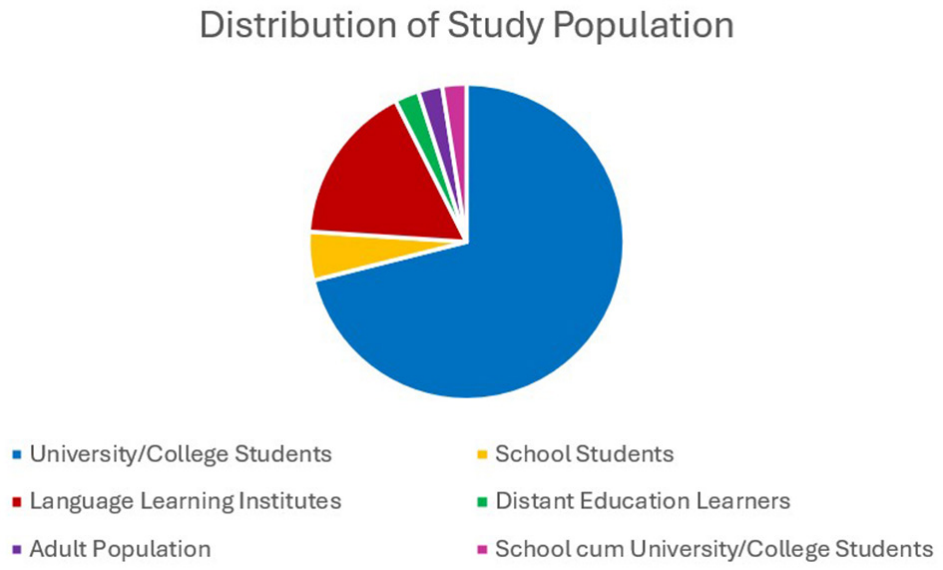
Population among whom the research have been conducted. The figure indicates that study population for majority of the studies are university/college students.

The study population for the majority (70%−84%) of the studies were university or college students. Approximately 17% (21) of the studies were conducted in language institutes. Seven studies (6%) were conducted among different groups: three on distant education learners, three on adult bilinguals and multilinguals, and one on pre-service teachers. However, limited research has been conducted on school students. Six (5%) studies were conducted among school students, and three (2%) were conducted among both school and university students. The distribution of study population across studies is depicted in Figure 8.

Evidently, 19 of the 69 studies in Iran were conducted among learners in language institutes. In Iran, English is taught in two ways: the public sector provides formal education, where English is not taught in primary education, and students are exposed to the language from the seventh grade. Alternatively, private sectors have established language centers with no age limit for enrolment (Moharami and Daneshfar, 2021). The quality of language material provided by public schools is not satisfactory (Torki and Chalak, 2017), and non-religious content in textbooks is censored by government officials (Benham and Mozaheb, 2013). Three studies compared two different study populations, two of which were conducted between school and university students, and the other compared online learners to in-person learners. There is limited research on primary school students because measuring EI in children is challenging due to their limited self-reflective capacity, developmental constraints in emotional understanding, and lack of psychometrically robust instruments (Denham et al., 2007; Matthews et al., 2012; Rivers et al., 2012).

### 3.4 Characteristics of studies on EI and ESL/EFL

**Figure 9:**
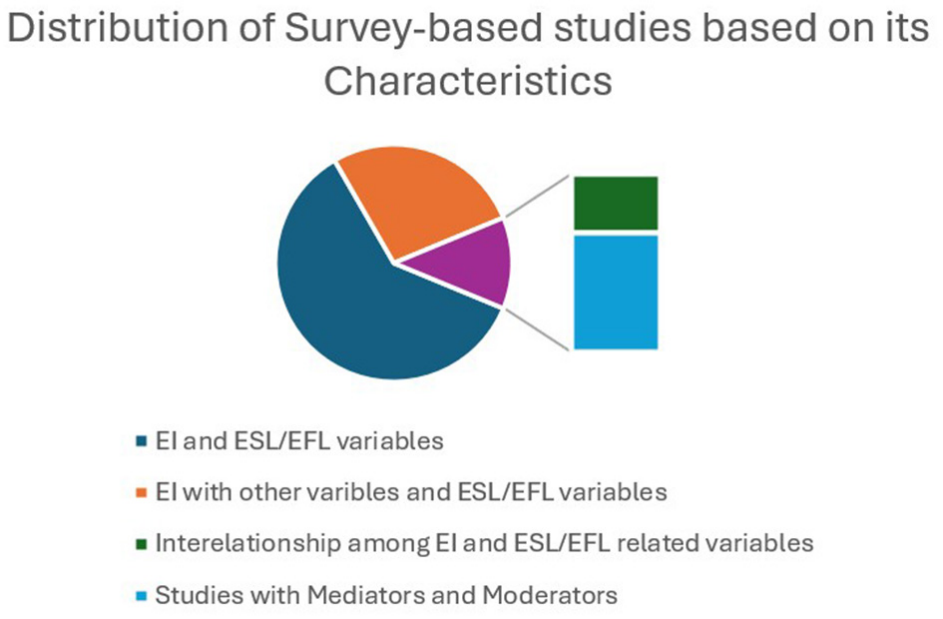
Distribution of survey-based studies. Numerous studies focus only on EI and other variables related to ESL/EFL learning.

This section analyses the characteristics, how EI has been explored with different variables of ESL/EFL learning, of the selected studies. The selected quantitative and qualitative descriptive studies can be classified into four types based on its characteristics. First, learners' EI was considered as an independent variable affecting ESL/EFL learning. Second, the influence of EI along with other variables (self-efficacy, self-esteem, classroom climate/environment) was examined using ESL/EFL-related variables (performance/achievement, learning strategy, willingness to communicate, anxiety, and enjoyment). Third, EI was viewed as a mediator between independent variables (teacher–student relationship, neuroticism, and FLA) and dependent variables (learning enjoyment, burnout, speaking achievement, and academic success). Fourth, studies that investigate the relationships among EI and other variables related to ESL/EFL learning. Figure 9 illustrates the distribution of survey-based research based on its characteristics.

**Figure 10:**
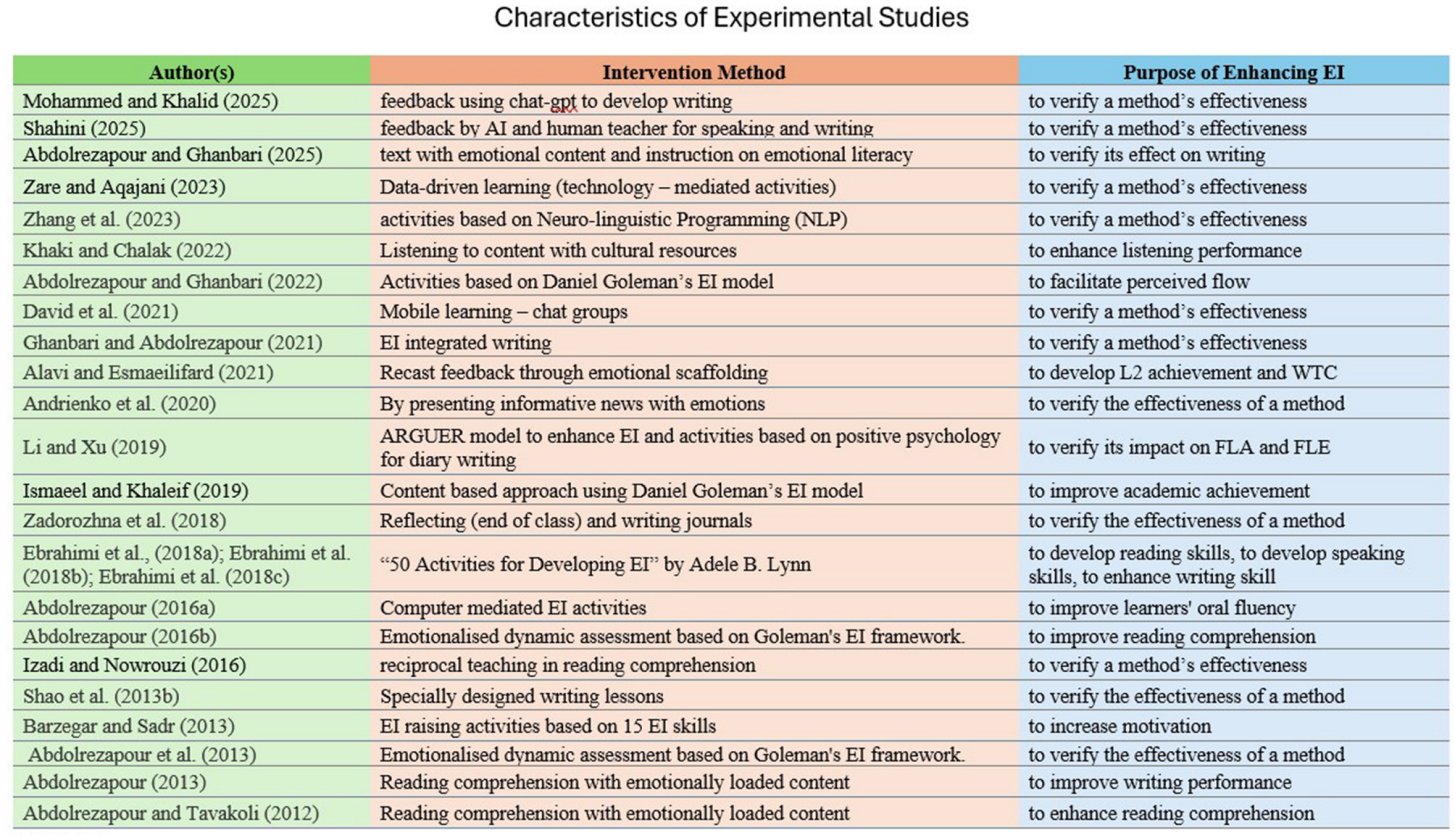
Intervention methods used to enhance EI in ESL/EFL classrooms and its purpose.

Furthermore, experimental research on EI and ESL/EFL learning can be divided into two types based on its purpose, although both aim to enhance EI. One type aims to examine how the enhancement of EI reflects on other variables related to ESL/EFL learning, whereas the second focuses on nurturing EI in ESL/EFL classrooms by analyzing the effect of the methods. Among the selected experimental studies, 16 focused on nurturing EI to develop competencies related to ESL/EFL learning. Of the selected articles (25), 17 were from Iran, and eight were from other countries. Although China has published a large number of articles in the past decade, only two experimental studies have been conducted so far. The characteristics of intervention studies are provided in Figure 10.

**Figure 11:**
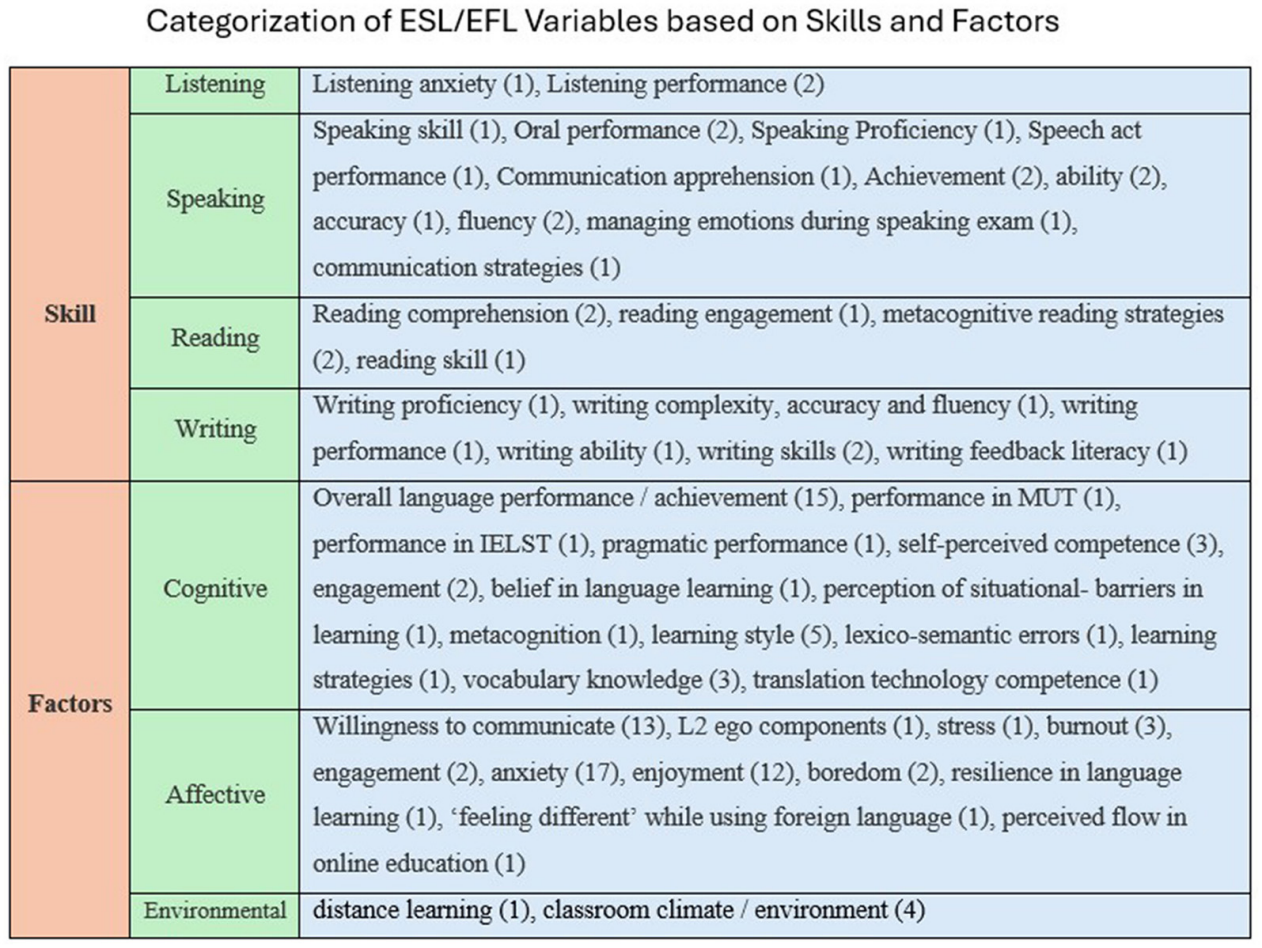
ESL/EFL variables investigated with EI has been categorized. EI has been investigated widely with variables associated with speaking skill and affective factors.

Depending on the nature of ESL/EFL variables investigated with EI, it has been classified into two broad types: variables related to skill and variables related to factors that influence language learning. Furthermore, skills associated variables were divided into listening, speaking, reading, and writing; whereas factors associated variables were classified into cognitive factors, affective factors and environmental factors. The details of these classifications are listed in Figure 11.

Categorizing the ESL/EFL variables investigated with EI based on skills and factors revealed that the impact, role, and effect of EI have been examined predominantly with variables associated with speaking skills. Speaking skills have been widely explored because they are considered an important of the four skills (Bueno et al., 2006); students' language proficiency is judged in real time through speaking skills (Brown, 2004), and it is the skill that the world demands which ESL/EFL teachers should focus on (Rao, 2019). Considering these factors, the cognitive and emotional factors of ESL/EFL learning are of equal importance. Among cognitive factors, overall performance/achievement has been extensively studied, and among affective factors, FLA (anxiety) and FLE (enjoyment) have been widely investigated among emotional factors. This finding supports Yu (2022) assertion that, in the last half-century, anxiety and enjoyment have been the two common emotions that have attracted most research.

### 3.5 Methodologies of studies on EI and ESL/EFL

#### 3.5.1 Data collection method

**Figure 12:**
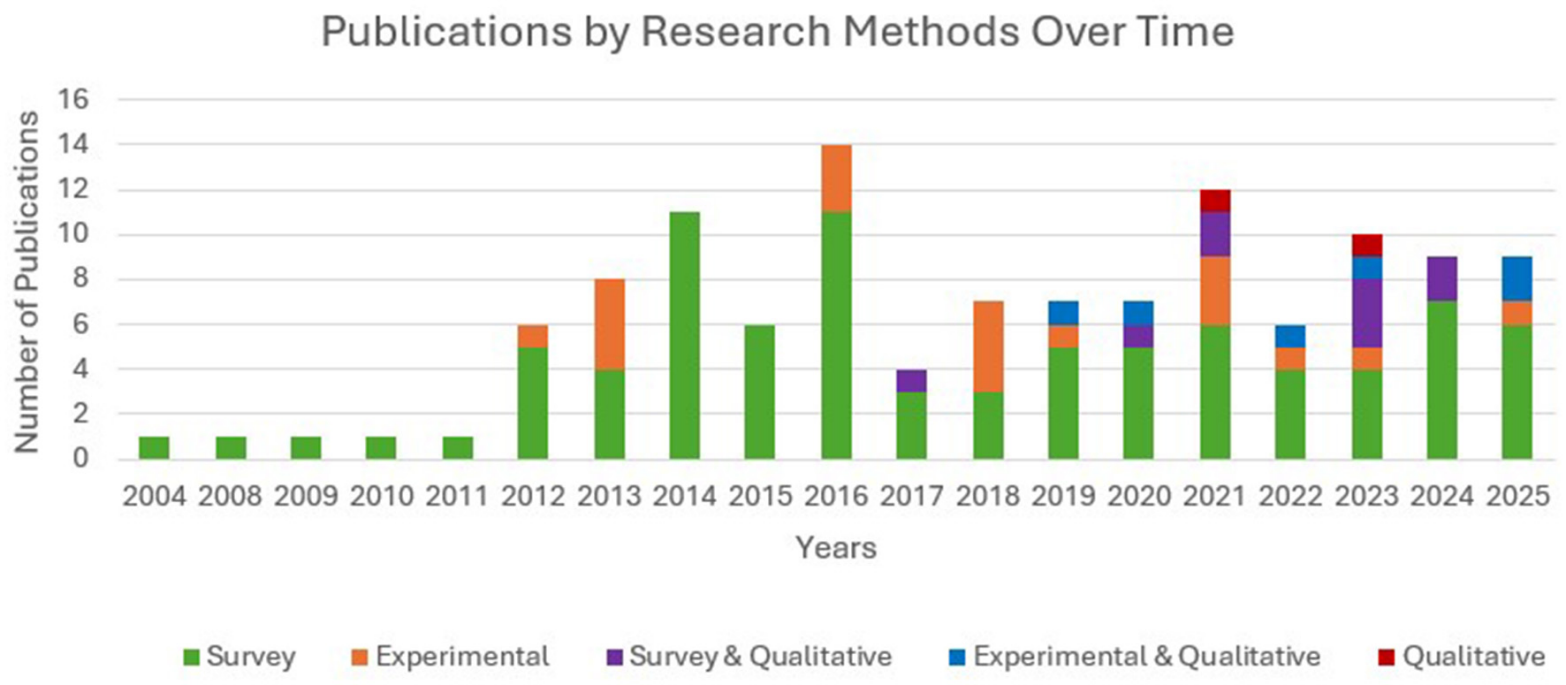
Year-wise publications based on research methods. Initially it was either a survey method or an experimental method. The figure indicates the evolution of research methods over time.

The methodology helps to verify the framed hypothesis of the research through design, data collection, and analysis. The relationship between EI and other variables of ESL/EFL learning can be studied through survey methods using appropriate data analysis tools. Furthermore, experimental research helps to prove the robustness of such relationships. Among the selected studies (121) for this review, 94 (78%) were survey-based studies, of which nine studies employed mixed methods (collected qualitative data along with surveys), 25 studies (20%) were experimental (intervention) studies, of which six studies employed mixed methods, and two studies (2%) were exclusively qualitative in nature. Figure 12 depicts the publication trends based on research methods over years.

**Figure 13:**
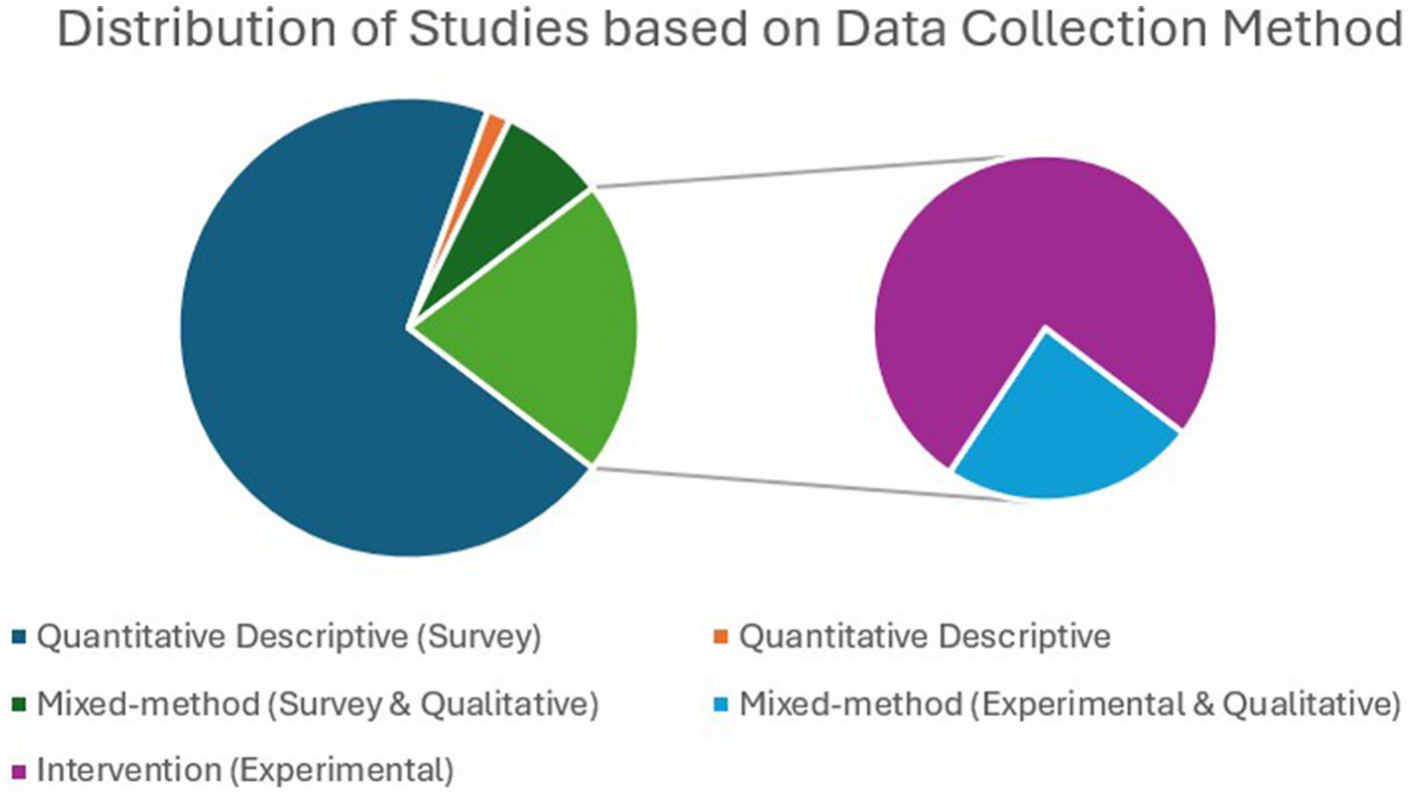
Distribution of data collection methods. Majority of the studies adopted survey-method for data collection.

Among the 14 studies that used a mixed-method design, three adopted an explanatory sequential design (Bata and Aqajani, 2023; Bata and Castro, 2021; Li and Xu, 2019). An explanatory sequential study collected quantitative data, and statistically significant results from the quantitative data were further validated, enriched, and embellished through qualitative findings (Creswell and Clark, 2017). Li and Xu (2019) combined two studies to study the relationship between EI and emotions (anxiety and enjoyment) in language classroom. The first study employed a survey method for quantitative data and open-ended questions for qualitative analyses. In the second study, quantitative data were collected from the intervention, followed by semi-structured interviews with teachers and participants to validate the quantitative findings. Bata and Castro (2021) studied the management of emotions during speaking exams, for which quantitative data were collected using the Trait Meta-Mood Scale to identify students with high and low EI levels, and qualitative data were collected through observation, open-ended questions, and interviews. According to Li and Dewaele (2021), the availability and ease of self-report EI measurement tools result in correlational and predictive designs rather than experimental designs that explore causal relationships. Moreover, EI is a stable trait, and designing interventions to enhance learners' EI levels in a short time frame is challenging (Mayer et al., 2008; Petrides, 2011). Figure 13 illustrates the distribution of various research methods.

#### 3.5.2 Methods used to measure EI

**Figure 14:**
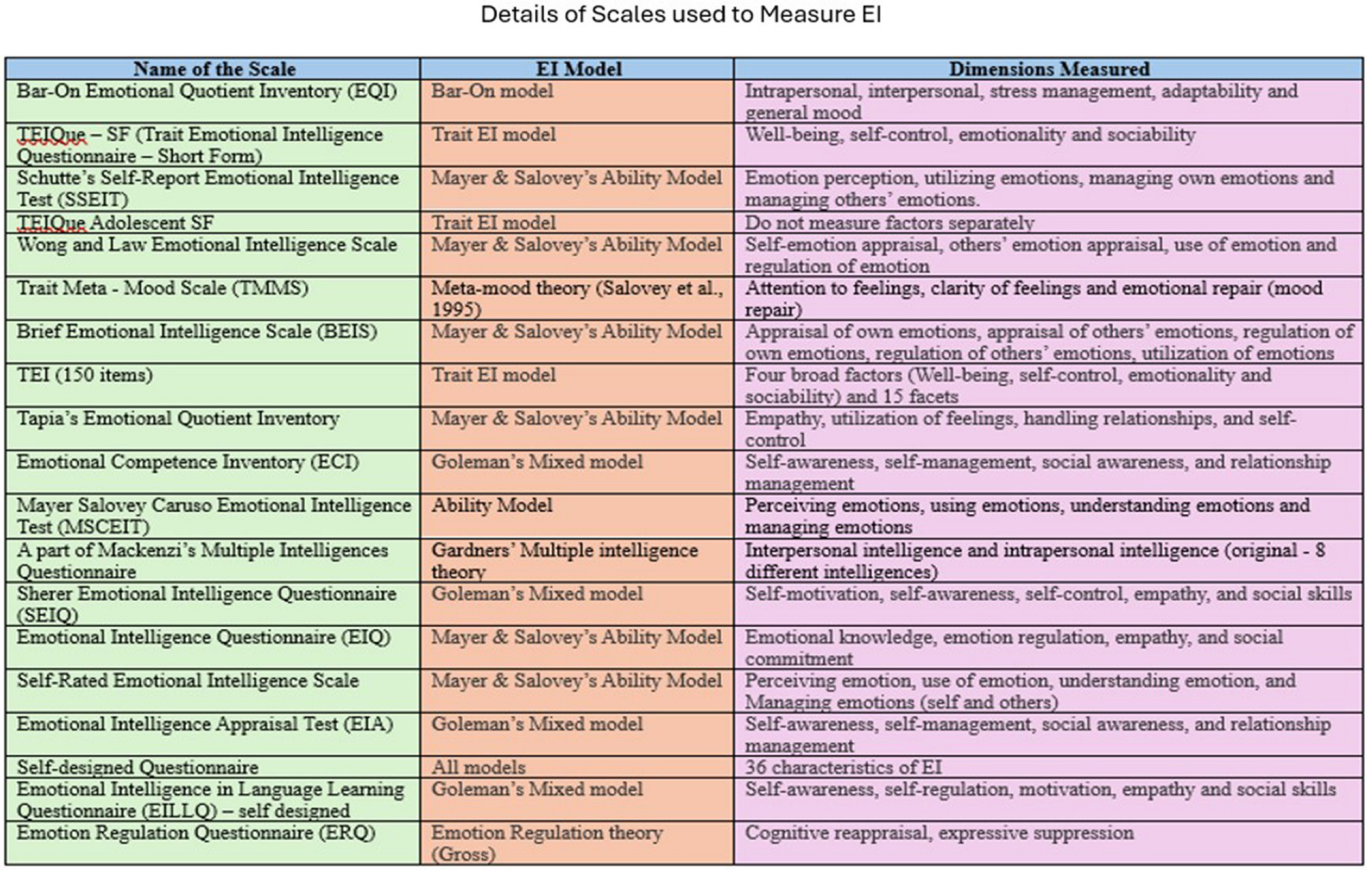
List of scales employed to measure EI along with EI model and dimensions measured.

Considering the method adopted to measure EI, thirty methods have been widely employed to measure EI (O'Connor et al., 2019). A self-report scale that assesses individuals' perception of emotional competence rather than actual behavior has been employed to measure EI in large-scale studies because of its ease of administration and practicality (Petrides and Furnham, 2000; Schutte et al., 1998; Siegling et al., 2015). Although a tool that measures performance, such as the MSCEIT (Mayer et al., 2000), exists, it is not frequently employed in educational contexts. Widely used scales to measure EI are the Bar-On Emotional Quotient Inventory (EQI) (33%) and Trait Emotional Intelligence–Short Form (TEIQue–SF) (30%). Among the 33% (40) of studies that employed the Bar-On EQI, 39 were from Iran and one was from Türkiye. Approximately 75% of the studies from China adopted either the TEIQue–SF or the Adolescent Short Form (TEIQue–ASF). However, by analyzing the EI measurement tools in the selected literature, it is evident that all but one study (Roohani, 2009) relied on self-report scales to measure EI. This finding indicates geographical differences in the EI measurements. Bar-On's (1997) EQ-*i*, based on a mixed model of EI, has been largely employed in the Iranian context; the TEIQue-ASF has been employed in eight studies, of which seven were conducted among learners from private language institutes in Iran and one among freshmen in China. The TEIQue-ASF was not used to measure EI in any of the four studies on school populations. In 2025, Wong and Law's Emotional Intelligence Scale was widely used in the Chinese context because it had only 16 items. The list of scales employed to measure EI, along with their theoretical models and dimensions measured, is listed in Figure 14.

#### 3.5.3 Data analysis method

**Figure 15:**
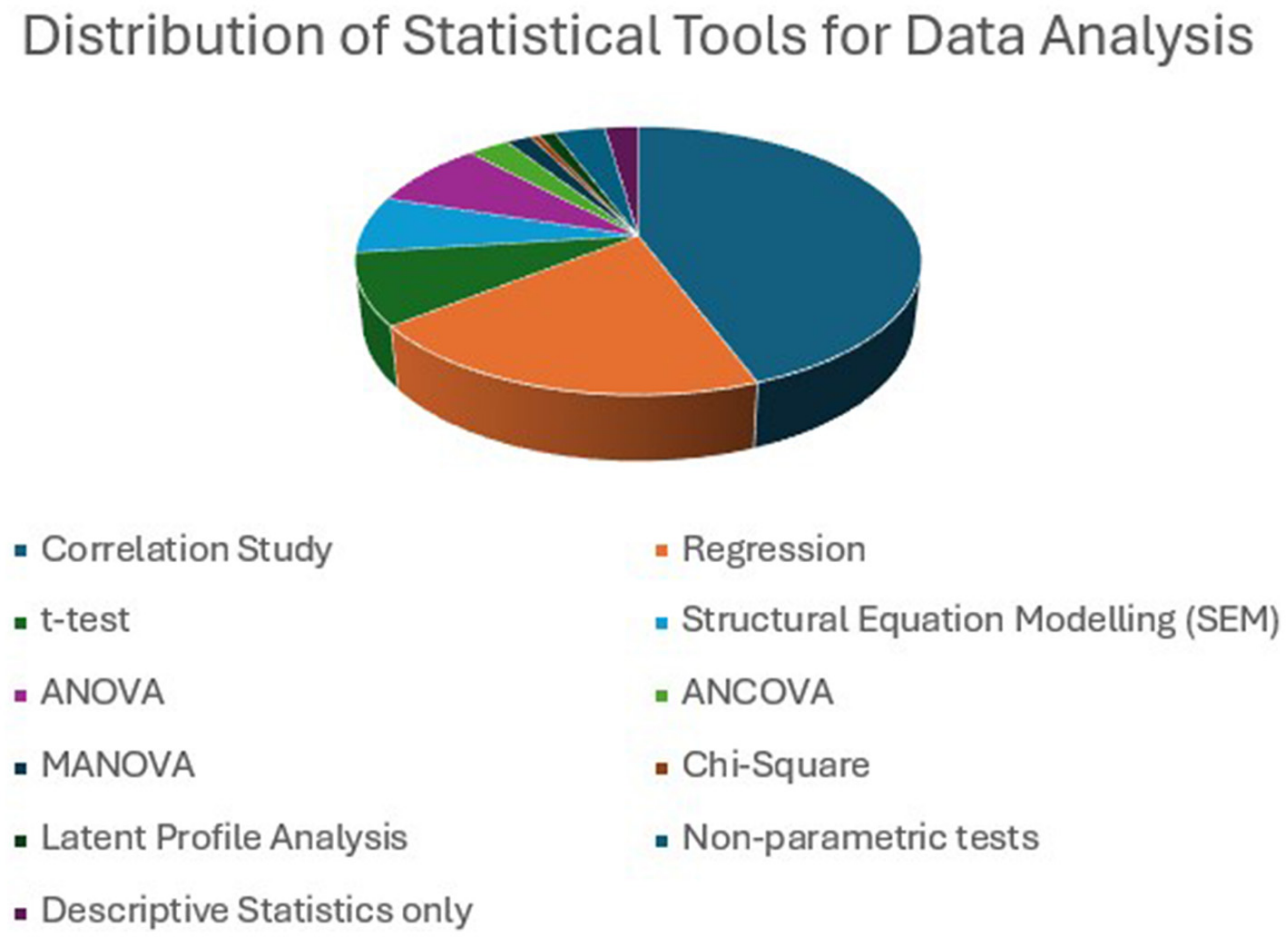
Different statistical tools employed for data analysis and its distribution. Correlation and regression are the widely used statistical tools.

Correlation (77) is a widely used statistical test for studying the relationship between EI and ESL/EFL variables. By examining the data analysis method, it is evident that the majority of research on EI and ESL/EFL primarily focuses on exploring the relationship between EI and other variables related to language learning. Along with this correlation, regression (35) was used to determine how strongly an independent variable, EI, predicts the dependent variables and constructs related to language learning. Comparatively, a few studies have analyzed the relationships between variables using ANOVA (14), ANCOVA (five), MANOVA (two), and SEM (12) tools. Paired sample *t*-tests were used in eight experimental studies to compare the differences between two groups (experimental and control groups) before and after the intervention. Methodological analysis further revealed that among the survey-based studies (94), 51 employed multivariate analysis, such as multiple regression, MANOVA, SEM, and LPA, and 43 employed bivariate analysis, including simple correlation, linear regression, and one-way ANOVA. SLL/FLL is a complex cognitive activity influenced by multiple emotions (Yu, 2022), and its relationship with EI can be better analyzed using MANOVA and SEM. Two recent studies (Gao et al., 2025; Long and Zhu, 2015) performed latent profile analysis by categorizing learners into four profiles based on EI level and studied how each profile possessed unique characteristics that further affected the variables of ESL/EFL learning. Figure 15 illustrates the distribution of various statistical tools employed across studies.

## 4 Findings and discussion

### 4.1 Key findings and discussion

This study makes a significant contribution to the field of EI and ESL/EFL research by conducting a systematic review of empirical studies, including quantitative descriptive, qualitative descriptive, mixed methods, and intervention studies. The publication trends in EI and ESL/EFL learning research have seen tremendous growth since 2016. Unlike other systematic reviews which predominantly focus on meta-analyses, this review used the TCCM framework to analyse and synthesize the theories, contexts, characteristics, and methodologies of research on EI and ESL/EFL learning. Analyzing the research through the TCCM framework helps to understand the evolution of different theoretical frameworks, contexts, characteristics, and methodologies over time. The findings of this review not only help to identify existing patterns in the TCCM context but also establish a roadmap for future studies.

The theoretical frameworks employed to understand the complex relationship between EI and ESL/EFL learning, study population and geographical location of the previous studies, and characteristics of the studies were analyzed to address RQ1 and achieve RO1. Considering theories, nearly 25% of the studies have theoretical underpinnings, and research on EI and ESL/EFL integrated theories has largely been conducted in the last 5 years (2021–2025). Examining the context revealed that a significant amount of research was conducted in Iran and China, where English is learnt as a foreign language, and the study population in the majority of the studies were ESL/EFL learners in university/college. In Iran, apart from school and university/college students, research has been conducted among learners from language learning institutes. Analyzing the studies' characteristics indicates that among the four skills, the impact and influence of EI has been widely investigated with speaking skill; and of the factors, the influence of EI has been predominantly studied with affective factors. These findings help to validate RA1, RA2, and RA3. Initially, it was assumed that diverse theoretical frameworks had been employed in the studies to understand the relationship and influence of EI in ESL/EFL learning; the results indicate that various theories have been integrated into the studies, particularly after 2020. It is evident that employing theories in EI and ESL/EFL learning research has evolved in the last 5 years (2021–2025). Based on RQ1 and RO1, it is also assumed (RA2) that research on EI and ESL/EFL learning has been conducted across different countries where English is learnt as a second/foreign language and among different study populations; however, the results reveal that a significant number of studies were conducted in countries where English is a foreign language and the study population for most of the studies is EFL learners in university/college. The third assumption (RA3) was also based on RQ1 and RO1. It was assumed that the impact of EI on all four language skills and the influence of EI on cognitive, affective, and environmental variables related to ESL/EFL have been widely investigated; however, the analysis revealed that EI has been widely investigated with variables related to speaking skill and affective factors in the ESL/EFL context. Less importance has been given to listening skills and environmental factors.

The different methods employed for data collection, various tools used to measure EI, and usage of several statistical tools for data analysis were analyzed to address RQ2 and achieve RO2. Based on RQ2 and RO2, it was assumed (RA4) that the use of data collection methods, measurement tools, and statistical tools has evolved significantly. Examining the research methods, the methods used to collect data revealed that numerous studies relied on quantitative descriptive (survey) methods. A single method, either the survey or intervention method, was used until 2016, and the trend of using mixed methods has evolved since 2017. However, only two studies have been based on qualitative data. Analyzing the tools employed to measure EI, it is evident that 99% of the studies measured EI using self-report scales based on different EI models. Considering the statistical tools employed for data analysis, most studies used either correlation or correlation and regression. The use of SEM for analysis has significantly increased since 2022, and two studies in 2025 used LPA. During the initial stage of research on EI and ESL/EFL learning, it is essential to understand the relationship between EI and other variables related to ESL/EFL, and how EI predicts language learning. Subsequently, the validation of such relationships resulted in an in-depth analysis which resulted in employing statistical tools such as ANOVA, ANCOVA, MANOVA, SEM, and LPA. It is evident that the usage of different statistical tools has evolved in the past 5 years (2021–2025).

### 4.2 Future scope using TCCM framework

#### 4.2.1 Theoretical scope

More than 75% of previous research on EI and ESL/EFL learning lacks a theoretical framework. Future researchers should employ theories in studies, especially quantitative studies, to strengthen their findings. Defining and agreeing upon a theoretical framework in an emerging field, such as EI and ESL/EFL, is quite challenging (Dewaele et al., 2020) because of its interdisciplinary nature. Emotional researchers can explore miscellaneous topics, approaches, and tools through the positive psychology approach, as it appreciates the plurality of empirical investigations and theories (MacIntyre et al., 2019). Future studies should focus on formulating new theories by drawing and integrating insights from other disciplines, such as general, educational, and social psychology (Dewaele et al., 2020) and qualitative research. Since emotions experienced by learners are dynamic in nature (Yu, 2022), EI can be further explored through the lens of complex dynamic systems theory to better understand the phenomenon and verify the fluctuations of emotions during different activities performed in a second/foreign language classroom.

#### 4.2.2 Contextual scope

Considering the countries, the existing literature on EI and ESL/EFL research was mostly conducted in Iran and China, where English is a foreign language. The results obtained from these studies cannot be generalized to countries where English is a second language because of the substantial differences in linguistic environments, educational practices, and cultural factors, which may influence EI and language learning dynamics. Comparative studies, such as Dewaele (2008), should be conducted across countries to understand the complex and dynamic relationship between EI and ESL/EFL learning. Of the study population, more than 90% of the study population are young adults and adults. Among the studies with school students as the study population, all were conducted among high school and secondary school students aged >12 years. No studies have been conducted on primary school students' (below 12 years) EI or their EFL/ESL. Future research should focus on primary school children because there are limited studies on the school population, particularly primary school children. Although it is challenging to measure EI among primary school children, methods other than self-report scales and questionnaires should be considered in future studies. A comparative study on the impact of socio-biographical variables on EI and ESL/EFL learning among primary, secondary, and tertiary level learners across different countries in Asia, Africa, the Middle East, and non-English speaking countries of Europe, North, and South America shall be a scope for further investigation.

#### 4.2.3 Characteristic scope

In the framework of EI and language skills, focus can be laid on EI and variables related to listening skills, as there are only limited studies (comprehension, attention span, memory/remembrance, and apprehension). In the context of emotional factors, Dewaele et al. (2020) stated that learners encounter different emotions, but anxiety and enjoyment have been well-studied in the context of second language acquisition. Although studies on anxiety and enjoyment persist, they have focused on classroom anxiety and enjoyment; however, anxiety and enjoyment during assessments are underexplored. Lo (2022) identified that enjoyment during language assessments promotes engagement and reduces anxiety; whereas how such positive emotions interact with EI competencies like self-awareness, self-regulation, and intrinsic motivation to influence language learning outcomes have to be investigated in future. Future researchers should focus on examining EI and the different emotions (positive and negative) experienced by ESL/EFL learners in the classroom, including skill-specific emotions. This suggestion aligns with Yu (2022), who stated that conducting comprehensive, cross-, and multi-factor research on the four skills can be a research agenda for future researchers, as the four language skills are not independent; they interact with each other to synthesize the overall experience and performance of learners.

#### 4.2.4 Methodological scope

Investigating EI in relation to ESL/EFL is a complex phenomenon which can be better performed using multiple methods of data collection and analysis. Numerous studies have been conducted using survey methods, whereas research based on intervention methods should be expanded to strengthen survey-based results. Comparative studies would be beneficial to compare the effects of different methods of nurturing EI in language classrooms, especially through AI and traditional methods. Mixed-method studies can be adopted in future research on EI and ESL/EFL learning, as the conceptualization of related constructs can be better performed through mixed-method design because 78% of the selected studies employed only quantitative methods. Among the different mixed-method designs, the exploratory sequential design can be used to conceptualize new constructs through qualitative data which can be further validated by quantitative data (Creswell, 2017). It can be used to design questionnaires or scales to measure variables. This method can be adopted by future researchers to conceptualize and operationalise different emotions related to ESL/EFL learning and to design a scale to measure them. As only two studies (Bata et al., 2021; Zhang, 2023) focus exclusively on qualitative methods of data collection, future studies should focus on qualitative data collection methods, such as focus group discussions, narrative enquiry, document analysis, and classroom observation, and emphasize case study design for in-depth and content-rich understanding. Furthermore, future researchers should focus on enhancing EI to facilitate academic literacy (Yang and Duan, 2023). Despite the existence of many instruments to measure EI, measuring it is challenging as it is a psychological construct and must rely on experts' opinions to define the correct answer (Roberts et al., 2001; Maul, 2012). In the future, EI and emotions in L2 classrooms can be measured through different means, such as the idiodynamic method, conversation analysis, clustering techniques of self-organizing maps, retrodictive qualitative modeling, and ecological momentary assessment based on the complex dynamic systems theory. Emotions were measured using self-report scales, and future physiological measures and behavioral observations can be performed to identify the intensity of emotions during different tasks in the SLL/FLL that can be analyzed using R packages. A review that exclusively analyzes EI measurement scales and their psychometric strengths/limitations would be a promising topic for future research. Statistical tools, such as ANOVA, ANCOVA, MANOVA, SEM, and LPA, in both survey and experimental methods, should be employed in the future to understand the complex relationship between EI and ESL/EFL learning.

### 4.3 Limitations of the study and future scope

This study reviews only empirical research, including quantitative descriptive studies, qualitative descriptive studies, intervention studies, and mixed methods that combine either quantitative descriptive (survey) and qualitative descriptive (observation, semi-structured interview, journal interpretation) or intervention (experimental) and qualitative descriptive (semi-structured interview) in the field of EI and ESL/EFL learning available in three databases: Scopus, Web of Science, and ERIC. It excluded research that focused on teachers' EI and its impact and role in ESL/EFL teaching and learning. This review analyzed the selected articles exclusively using the TCCM framework; however, the results and findings of these studies were not discussed. This study synthesizes the theoretical frameworks integrated into the studies, different contexts in which the research has been conducted, characteristics of the studies, and various methods used for data collection, EI measurement, and data analysis, and discusses how it has evolved over the years. However, attention has not been given to in-depth analysis of each TCCM components.

In the future, the TCCM framework can be applied to reviews of ESL/EFL teachers' EI and its impact on teaching and learning. A bibliometric analysis of the same topic helps to better understand the contributions of different countries to the field. As this review does not emphasize statistical values, a meta-analysis of the impact of EI on different variables is a promising scope for future research. Since this study is limited to only ESL/EFL, future reviews on EI and its impact on SLL/FLL which include other languages, can be conducted. The impact of EI on SLL/FLL may vary depending on linguistic and cultural differences. An exclusive review of intervention studies which focus on enhancing EI in ESL/EFL classrooms helps to identify the possible methods to nurture EI and their effects. Each component of TCCM should be reviewed independently for a deep understanding of theoretical frameworks, contexts, study characteristics, and methodologies of the studies which in turn helps to identify the evolution and common patterns paving the way to integrate novelties in future research by all possible means.

## 5 Conclusion

This systematic literature review synthesized and analyzed 121 research documents on EI and ESL/EFL learning selected from the Scopus, Web of Science and ERIC databases and provides a research agenda for future studies based on TCCM framework. Examining the studies based on the TCCM framework helps to identify the predominant theoretical frameworks integrated into the studies, locate the geographical context and study populations of the majority of the studies, classify the studies based on their characteristics, and examine the different methods used for data collection, EI measurement, and data analysis. Subsequently, the evolution of each TCCM component over the years was observed. Among the selected articles, 23% integrated theoretical frameworks, and the broaden-and-build theory of positive psychology and control–value theory of achievement emotions were widely employed. Context analysis indicated that 17 countries contributed to the field, with a substantial body of literature (74%) from Iran and China. Within the context of the study population, extensive literature is available on university/college/tertiary-level learners; however, research on school students remains limited. Six studies were conducted among high/senior school students aged above 12 years. Examining the characteristics of the study indicates that EI has been extensively investigated in relation to speaking skills and emotional factors such as anxiety and enjoyment in ESL/EFL learning. Approximately 78% of the studies adopted the survey method to explore the relationships between EI and variables related to ESL/EFL learning, while 25 studies focused on enhancing EI in ESL/EFL classrooms. Scrutinizing the methodologies indicates that 15 studies adopted a mixed-method design to study the phenomena, all the studies implemented self-assessment tools to measure EI, and the predominant statistical tools employed in the existing literature are correlation and regression analyses.

The findings of this review evidently indicate a significant evolution in the patterns of all TCCM components. Underexplored areas and potential ways to address them in future research are elucidated. The limitations of this study are that it relied only on three databases, there exists a risk of bias assessment in the selection of studies because it lacks dual independent screening and no formal tools have been employed to assess the methodological quality, it has not examined studies other than ESL/EFL, and the statistical values of the studies were not included in the analysis. This review analyzed the prevailing literature using the TCCM framework. Future researchers should consider bibliometric analyses, meta-analyses, and scoping reviews by expanding the databases. A meta-analysis of EI and its impact on the four language skills would be a promising topic for future studies. Future researchers should design activities that nurture EI in a manner that can be integrated into language teaching. Despite its limitations, this study offers significant contributions by disseminating knowledge on theoretical frameworks, demographic profiles of study populations and countries, features of the studies, and methodologies adopted in the existing literature on EI and ESL/EFL learning.

## Data Availability

The original contributions presented in the study are included in the article/Supplementary material, further inquiries can be directed to the corresponding author.
