# Supplementary material for: Emotional intelligence and English as a second/foreign language learning: a systematic review using TCCM framework
Source: Front Psychol. 2026 Jan 9;16:1722555. doi: 10.3389/fpsyg.2025.1722555 (PMC12827750; doi:10.3389/fpsyg.2025.1722555)
Supplement: Supplementary file 1 [file Supplementary_file_1.docx]

**EMOTIONAL INTELLIGENCE AND ENGLISH AS A SECOND/FOREIGN LANGUAGE LEARNING: A SYSTEMATIC REVIEW USING TCCM FRAMEWORK**

G. Deepika^1^, J. Mary Jennifer*2

1. Research Scholar, Department of English, School of Social Sciences and Languages, Vellore Institute of Technology, Vellore, India.

2. Assistant Professor, Department of English, School of Social Sciences and Languages, Vellore Institute of Technology, Vellore, India.

*Corresponding author’s email: maryjennifer.j@vit.ac.in

**References of Analysed Studies**

*Abdolrezapour, P., and Tavakoli, M. (2012). The relationship between emotional intelligence and EFL learners’ achievement in reading comprehension. *Innovation in Language Learning and Teaching*, *6*(1), 1-13. <https://doi.org/10.1080/17501229.2010.550686>

*Abdolrezapour, P., Tavakoli, M., and Ketabi, S. (2013). Enhancing learners’ emotions in an L2 context through emotionalized dynamic assessment. *Studies in Second Language Learning and Teaching*, *3*(2), 213. <https://doi.org/10.14746/ssllt.2013.3.2.4>

*Abdolrezapour, P. (2013). The relationship between emotional intelligence and EFL learners’ writing performance. *Procedia - Social and Behavioral Sciences*, *70*, 331-339. <https://doi.org/10.1016/j.sbspro.2013.01.070>

*Abdolrezapour, P. (2016a). Improving learners’ oral fluency through computer-mediated emotional intelligence activities. *ReCALL*, *29*(1), 80-98. <https://doi.org/10.1017/S0958344016000069>

*Abdolrezapour, P. (2016b). Improving L2 reading comprehension through Emotionalized dynamic assessment procedures. *Journal of Psycholinguistic Research*, *46*(3), 747-770. <https://doi.org/10.1007/s10936-016-9464-9>

*Abdolrezapour, P. (2018). The relationship between emotional intelligence and complexity, accuracy, and fluency in EFL Learnersâ€™ oral performance. *Cypriot Journal of Educational Sciences*, *13*(3), 310-318. <https://doi.org/10.18844/cjes.v13i3.3452>

*Abdolrezapour, P., and Ghanbari, N. (2022). Emotional-based pedagogy and facilitating EFL learners' perceived flow in online education. *Frontiers in Psychology*, *13*. <https://doi.org/10.3389/fpsyg.2022.960287>

*Abdolrezapour, P., and Ghanbari, N. (2025). The impact of an emotional literacy intervention on students’ composing processes in EFL integrated writing tasks. *The Language Learning Journal*, *53*(3), 372-388. <https://doi.org/10.1080/09571736.2025.2473477>

*Afshar, H. S., and Rahimi, M. (2014). The relationship among critical thinking, emotional intelligence, and speaking abilities of Iranian EFL learners. *Procedia - Social and Behavioral Sciences*, *136*, 75-79. <https://doi.org/10.1016/j.sbspro.2014.05.291>

*Afshar, H. F., Tofighi, S., and Hamazawi, R. (2016). Iranian EFL learners' emotional intelligence, learning styles, strategy use, and their L2 achievement. *Issues in Educational Research*, *26*(4), 635 - 652.

*Alavi, S. M., and Esmaeilifard, F. (2021). The effect of emotional scaffolding on language achievement and willingness to communicate by providing recast. *Cogent Psychology*, *8*(1). <https://doi.org/10.1080/23311908.2021.1911093>

* Alavinia, P., and Alikhani, M. A. (2014). Willingness to communicate reappraised in the light of emotional intelligence and gender differences. *Procedia - Social and Behavioral Sciences*, *98*, 143-152. <https://doi.org/10.1016/j.sbspro.2014.03.400>

*Alavinia, P., and Behyar, N. (2012). On the feasible linkages between Iranian academic EFL Learners‟ emotional intelligence level and their Lexico-semantic errors in writing. *Theory and Practice in Language Studies*, *2*(6). <https://doi.org/10.4304/tpls.2.6.1174-1184>

*Alavinia, P., and Ebrahimpour, S. (2012). On the correlation between emotional intelligence and learning styles: The case of Iranian academic EFL learners. *Theory and Practice in Language Studies*, *2*(6). <https://doi.org/10.4304/tpls.2.6.1291-1299>

*Alavinia, P., and Mollahossein, H. (2012). On the Correlation between Iranian EFL Learners' Use of Metacognitive Listening Strategies and Their Emotional Intelligence. *International Education Studies*, *5*(6), 189-203. <https://doi.org/10.5539/ies.v5n6p189>

*Algraini, F. N. (2022). The relationship of Metacognitive reading strategies used by Saudi EFL learners and their emotional intelligence. *International Journal of English Language and Literature Studies*, *11*(1), 42-56. <https://doi.org/10.55493/5019.v11i1.4440>

*Aliasin, S. H., and Abbasi, S. (2020). The relationship between Iranian EFL learners’ emotional intelligence and Metacognitive reading strategies use. *Journal of Language and Education*, *6*(2), 31-43. <https://doi.org/10.17323/jle.2020.9730>

*Aljasir, N. (2024). Emotional intelligence and engagement in language learning: A mixed-methods exploration of their impact on English language proficiency. *Theory and Practice in Language Studies*, *14*(9), 2982-2995. <https://doi.org/10.17507/tpls.1409.34>

*Alrabai, F., and Alamer, A. (2022). The role of learner character strengths and classroom emotions in L2 resilience. *Frontiers in Psychology*, *13*. <https://doi.org/10.3389/fpsyg.2022.956216>

*Amini, F. A., and Sabber, Z. (2015). On the Relationship between Willingness to Communicate and Emotional Intelligence: a Case of Iranian EFL Learners. *Modern Journal of Language Teaching Methods*, *5*(3), 36.

*Andrienko, T., Chumak, N., and Genin, V. (2020). Emotional intelligence and acquisition of English language oral communication skills. *Advanced Education*, *7*(15), 66-73. <https://doi.org/10.20535/2410-8286.201013>

*Atapour, Z. (2016). The Relationship between Iranian EFL Learners' Emotional Intelligence and their Learning Styles (Reflectivity/Impulsivity). *Modern Journal of Language Teaching Methods*, *6*(1), 108.

*Bagheri, M. S., and Ghasemi, E. (2013). The relationship between emotional intelligence and writing performance of IELTS learners. *Modern Journal of Language Teaching Methods*, *3*(4), 83.

*Barzegar, R., and Sadr, S. A. (2013). The effect of emotional intelligence awareness-raising activities on L2 motivation. *Studies in Second Language Learning and Teaching*, *3*(1), 67. <https://doi.org/10.14746/ssllt.2013.3.1.4>

*Bata, S., and Castro, C. (2021). English as a Foreign Language Students’ Emotional Intelligence Management When Taking Speaking Exams. *Profile Issues in Teachers` Professional Development*, *3*(2), 245 - 261. <https://doi.org/10.15446/profile.v23n2.88378>

*Chen, Z., and Zhang, P. (2020). Trait emotional intelligence and second language performance: A case study of Chinese EFL learners. *Journal of Multilingual and Multicultural Development*, *43*(8), 731-745. <https://doi.org/10.1080/01434632.2020.1767633>

*Chen, Z., Zhang, P., Lin, Y., and Li, Y. (2021). Interactions of trait emotional intelligence, foreign language anxiety, and foreign language enjoyment in the foreign language speaking classroom. *Journal of Multilingual and Multicultural Development*, *45*(2), 374-394. <https://doi.org/10.1080/01434632.2021.1890754>

*Dastgoshadeh, A., and Javanmardi, P. (2021). Emotional Intelligence as a Predictor of EFL Learners' Willingness to Communicate. *Mextesol Journal*, *45*(3), n3.

*David, I., Eka, W., Areta, P., Mutiara, B., and Rizki, F. A. (2021). An experiment on mobile learning to leverage EFL learners’ engagement, emotional intelligence, and learning motivation. *The Journal of Asia TEFL*, *18*(4), 1285-1301. <https://doi.org/10.18823/asiatefl.2021.18.4.13.1285>

*Dewaele, J.M, Petrides, K. V., and Furnham, A. (2008). Effects of trait emotional intelligence and Sociobiographical variables on communicative anxiety and foreign language anxiety among adult multilinguals: A review and empirical investigation. *Language Learning*, *58*(4), 911-960. <https://doi.org/10.1111/j.1467-9922.2008.00482.x>

*Ebrahimi, M. R., Khoshsima, H., and Zare-Behtash, E. (2018a). The impacts of enhancing emotional intelligence on the development of reading skill. *International Journal of Instruction*, *11*(3), 573-586. <https://doi.org/10.12973/iji.2018.11339a>

*Ebrahimi, M. R., Khoshsima, H., Zare-Behtash, E., and Heydarnejad, T. (2018b). Emotional intelligence enhancement impacts on developing speaking skill among EFL learners: An empirical study. *International Journal of Instruction*, *11*(4), 625-640. <https://doi.org/10.12973/iji.2018.11439a>

*Ebrahimi, M. R., Khoshsima, H., and Zare-Behtash, E. (2018c). The impacts of emotional intelligence enhancement on Iranian intermediate EFL learners writing skill. *International Journal of Instruction*, *11*(1), 437-452. <https://doi.org/10.12973/iji.2018.11130a>

*Farahani, A. A., and Gholam-Shahbazi, H. (2019). The relationship between the emotional intelligence of Iranian EFL learners and their performance on the listening section of IELTS. *Journal of Language Teaching and Research*, *10*(3), 469. <https://doi.org/10.17507/jltr.1003.09>

*Farsad, L., and Modarresi, G. (2023). EFL learners’ construction of L2 ego and its relationship with emotional intelligence. *Journal of Research in Applied Linguistics*, *14*(1), 124-139. <https://doi.org/10.22055/rals.2023.18072>

*Gao, Y., Guo, Y., and Wang, Y. (2025). Chinese English as a foreign language college students’ emotional intelligence and willingness to communicate: A latent profile analysis. *Perceptual and Motor Skills*, *132*(1), 119-143. <https://doi.org/10.1177/00315125241283151>

* Gao, Z., and Yang, Y. (2023). The predictive effect of trait emotional intelligence on emotion regulation strategies: The mediating role of negative emotion intensity. *System*, *112*, 102958. <https://doi.org/10.1016/j.system.2022.102958>

*Ghanadi, Z., and Ketabi, S. (2014). The relationship between emotional intelligence and learners’ beliefs about language learning: Iranian advanced EFL learners in focus. *Theory and Practice in Language Studies*, *4*(3). <https://doi.org/10.4304/tpls.4.3.518-523>

*Ghabanchi, Z., and Rastegar, R. E. (2014). The correlation of IQ and emotional intelligence with reading comprehension. *Reading Matrix*, *14*(2), 135-144.

*Ghaemi, H., and Kodabakhsh, M. (2016). The Relationship Between EFL Learners' Level of Reading Engagement and Their Emotional Intelligence. *Modern Journal of Language Teaching Methods*, *6*(1), 407.

*Ghanbari, N., and Abdolrezapour, P. (2021). Using emotional intelligence in an EFL integrated writing assessment. *Studies in Educational Evaluation*, *70*, 101017. <https://doi.org/10.1016/j.stueduc.2021.101017>

*Ghonsooly, B., and Mazaheri, H. (2010). On the relationship between emotional intelligence and directive speech acts preference. *Journal of Research in Applied Linguistics*, *1*(1), 50-68. <https://rals.scu.ac.ir/article_10414.html>

* Hamdzah, N. L., Subramaniam, I. D., Abidin, N. Z., and Hassan, R. (2020). The relationship between emotional intelligence (EI) and the Malaysian University English test (MUET) performance among technical students. *International Journal of Learning, Teaching and Educational Research*, *19*(7), 280-297. [*https://doi.org/10.26803/ijlter.19.7.16*](https://doi.org/10.26803/ijlter.19.7.16)

* Hamidrez, K., and Maryam, F. (2016). *The Relationship Between EI (Emotional Intelligence) and Speaking Proficiency in e-Learning*. In *ECEL 2016 - Proceedings of the 15th European conference on e- Learning*. Academic Conferences and publishing limited.

* Han, S., Li, Y., and Haider, S. A. (2022). Impact of foreign language classroom anxiety on higher education students academic success: Mediating role of emotional intelligence and moderating influence of classroom environment. *Frontiers in Psychology*, *13*. <https://doi.org/10.3389/fpsyg.2022.945062>

*Imamyartha, D., Widiati, U., and Anugerahwati, M. (2023). The nexus between emotional intelligence, learning engagement, motivation, and achievement in team-based mobile language learning. *The JALT CALL Journal*, *19*(2), 269-298. <https://doi.org/10.29140/jaltcall.v19n2.1083>

*Ismaeel, A. A., and Khaleif, K. S. (2019). The interrelatedness between the content-based approach and EFL University students' emotional intelligence. *Opción: Revista de Ciencias Humanas y Sociales*, (22), 319-330.

* Izadi, M., and Nowrouzi, H. (2016). Reciprocal teaching and emotional intelligence: A study of Iranian EFL learners’ reading comprehension. *The Reading Matrix: An International Online Journal*, *16*(1), 133-147.

*Jalilzadeh, K., and Yeganehpour, P. (2021). The relationship between intermediate EFL students’ oral performance, communicative willingness, as well as emotional intelligence. *The Reading Matrix: An International Online Journal*, *21*(2), 29-48.

*Jin, F., Gu, C., and Li, Y. (2024). Trait emotional intelligence and foreign language performance: Associations with academic self-efficacy and foreign language anxiety. *Frontiers in Education*, *9*. <https://doi.org/10.3389/feduc.2024.1419328>

*Karimi, G. (2012). How Do Productive Skills of L2 Learning Require EI?. In *International Conference on Languages, E-Learning and Romanian Studies*.

*Karimi, M. N. K., Hashemi, M. R., and Sarbazfard, M. (2016). Emotional intelligence and critical thinking ability as correlates of EFL learners’ vocabulary knowledge. *Journal of Research in Applied Linguistics*, *7*(1), 75-94. <https://doi.org/10.22055/rals.2016.11778>

*Khademi, H., and Farokhmehr, M. (2016, October). The Relationship between EI (Emotional Intelligence) and speaking proficiency in e-Learning. In *ECEL 2016-Proceedings of the 15th European Conference on e-Learning* (p. 357). Academic Conferences and publishing limited.

*Khaghaninejad, M. S., Moloodi, A. S., and Shojaee, S. (2017). How are EQ, IQ and Self-esteem related to Reading Comprehension of Intermediate and Advanced Iranian EFL Learners?. *Modern Journal of Language Teaching Methods*, *7*(6), 48-63.

*Khaki, M., and Chalak, A. (2022). Cultural resources, listening comprehension of intermediate Iranian EFL learners and their emotional intelligence. *Language Testing in Focus: An International Journal*, *5*, 5-13. <https://doi.org/10.32038/ltf.2022.05.02>

*Korpi, S., and Farvardin, M. T. (2016). Investigating the relationship between emotional intelligence and writing complexity, accuracy and fluency among graduate students of TEFL. *Modern Journal of Language Teaching Methods*, *6*(1). [234-243](https://www.citefast.com/234-243)

* Li, C. (2019). A positive psychology perspective on Chinese EFL students’ trait emotional intelligence, foreign language enjoyment and EFL learning achievement. *Journal of Multilingual and Multicultural Development*, *41*(3), 246-263. <https://doi.org/10.1080/01434632.2019.1614187>

* Li, C., Huang, J., and Li, B. (2021). The predictive effects of classroom environment and trait emotional intelligence on foreign language enjoyment and anxiety. *System*, *96*, 102393. <https://doi.org/10.1016/j.system.2020.102393>

*Li, G., and Pan, Y. (2025). Understanding trait emotional intelligence and L2 willingness to communicate: The serial mediation of perceived teacher support and L2 emotions. *Acta Psychologica*, *260*. <https://doi.org/10.1016/j.actpsy.2025.105539>

*Li, C., and Xu, J. (2019). Trait emotional intelligence and classroom emotions: A positive psychology investigation and intervention among Chinese EFL learners. *Frontiers in Psychology*, *10*. <https://doi.org/10.3389/fpsyg.2019.02453>

* Li, Y., and Zhang, L. (2024). Exploring the relationships among teacher–student dynamics, learning enjoyment, and burnout in EFL students: The role of emotional intelligence. *Frontiers in Psychology*, *14*. <https://doi.org/10.3389/fpsyg.2023.1329400>

*Long, Z., and Zhu, R. (2025). A latent profile analysis of emotional intelligence and its relationship with L2 student writing feedback literacy. *System*, *133*, 103773. [i.org/10.1016/j.system.2025.103773](https://doi.org/10.1016/j.system.2025.103773)[Get rights and content](https://s100.copyright.com/AppDispatchServlet?publisherName=ELS&contentID=S0346251X25001836&orderBeanReset=true)

*Mahasneh, A. M. (2014). Investigating the relationship between emotional intelligence and meta-cognition among Hashemite University students. *Review of European Studies*, *6*(4). <https://doi.org/10.5539/res.v6n4p201>

*Manzouri, H and Movahed, R (2017). Investigation of the relationship among Emotional Intelligence, Foreign Language Anxiety, Language Proficiency, and Achievement of Iranian EFL Learners. Revista Quid (Special Issue), 2464-2472.

*McEown, K., McEown, M. S., and Oga-Baldwin, W. L. (2023). The role of trait emotional intelligence in predicting academic stress, burnout, and engagement in Japanese second language learners. *Current Psychology*, *43*(2), <https://doi.org/10.1007/s12144-023-04296-8>

*Mede, E., and Budak, T. (2021). Relationship between emotional intelligence, foreign language anxiety, and Demotivational factors in an English preparatory language program. *Colombian Applied Linguistics Journal*, *24*(1), 6-22. <https://doi.org/10.14483/22487085.17859>

*Mehrpoor, S., and Soleimani, N. (2018). On the relationships among EFL learners willingness to communicate, Communication Apprehension, self-perceived competence and emotional intelligence. *Khazar Journal of Humanities and Social Sciences*, *21*(3), 5-20. <https://doi.org/10.5782/2223-2621.2018.21.3.5>

*Méndez López, M. G., and Bautista Tun, M. (2017). Motivating and demotivating factors for students with low emotional intelligence to participate in speaking activities. *PROFILE Issues in Teachers' Professional Development*, *19*(2), 151-163. <https://doi.org/10.15446/profile.v19n2.60652>

*Mohammadi, Z., and Izadpanah, S. (2018). The effect of emotional intelligence and gender on writing proficiency of Iranian EFL learners. *Journal of Language Teaching and Research*, *9*(1), 164. <https://doi.org/10.17507/jltr.0901.21>

*Mohammed, S. J., and Khalid, M. W. (2025). Under the world of AI-generated feedback on writing: Mirroring motivation, foreign language peace of mind, trait emotional intelligence, and writing development. *Language Testing in Asia, 15*(7). <https://doi.org/10.1186/s40468-025-00343-2>

*Mohazabieh, S., and Sadighi, F. (2016). The Relationship Between Emotional Intelligence, and Vocabulary Learning Strategies of Iranian EFL Learners. *Modern Journal of Language Teaching Methods*, *6*(4), 208.

*Mujiono, M., Nakhrowi, N., and Fatimah, S. (2019). The effect of verbal-linguistic intelligence and emotional intelligence on academic achievement of Indonesian EFL learners. *International Journal of Learning, Teaching and Educational Research*, *18*(12), 350-365. <https://doi.org/10.26803/ijlter.18.12.20>

* Namaziandost, E., Kargar Behbahani, H., and Heydarnejad, T. (2024). Tapping the alphabets of learning-oriented assessment: Self-assessment, classroom climate, mindsets, trait emotional intelligence, and academic engagement are in focus. *Language Testing in Asia*, *14*(1). <https://doi.org/10.1186/s40468-024-00293-1>

*Niroomand, S. M., Behjat, F., and Rostampour, M. (2014). A quantitative study on the relationship between EFL university student's emotional intelligence and motivation. *Modern Journal of Language Teaching Methods*, *4*(2), 137

*Nesari, A. J., Karimi, L., and Filinezhad, N. (2011). On the relationship between emotional intelligence and vocabulary learning of Iranian Efl learners at the intermediate level. *Procedia - Social and Behavioral Sciences*, *28*, 900-903. <https://doi.org/10.1016/j.sbspro.2011.11.165>

* Oz, H. (2015). Emotional intelligence as a predictor of L2 communication. *Procedia - Social and Behavioral Sciences*, *186*, 424-430. <https://doi.org/10.1016/j.sbspro.2015.04.117>

*Ożańska-Ponikwia, K., Piechurska-Kuciel, E., and Skałacka, K. (2020). Emotional intelligence as a mediator in the relationship between neuroticism and L2 achievement. *Applied Linguistics Review*, *14*(1), 67-86. <https://doi.org/10.1515/applirev-2020-0008>

*Ożańska-Ponikwia, K. (2012). What has personality and emotional intelligence to do with ‘feeling different’ while using a foreign language? *International Journal of Bilingual Education and Bilingualism*, *15*(2), 217-234. <https://doi.org/10.1080/13670050.2011.616185>

*Rahimi Domakani, M., Mirzaei, A., and Zeraatpisheh, S. (2014). L2 learners’ affect and pragmatic performance: A focus on emotional intelligence and gender dimensions. *Journal of Research in Applied Linguistics*, *5*(2), 149-174.

*Resnik, P., and Dewaele, J. (2020). Trait emotional intelligence, positive and negative emotions in first and foreign language classes: A mixed-methods approach. *System*, *94*, 102324. <https://doi.org/10.1016/j.system.2020.102324>

*Resnik, P., and Dewaele, J. (2021). Learner emotions, autonomy and trait emotional intelligence in ‘in-person’ versus emergency remote English foreign language teaching in Europe. *Applied Linguistics Review*, *14*(3), 473-501. [https://doi.org/10.1515/applirev‑2020‑0096](https://doi.org/10.1515/applirev20200096)

*Resnik, P., Moskowitz, S., and Panicacci, A. (2021). Language learning in crisis mode: The connection between LX grit, trait emotional intelligence and learner emotions. *The Journal for the Psychology of Language Learning*, *3*(2), 99-117.

*Resnik, P., Panicacci, A., and Dewaele, J. M. (2025). How trait emotional intelligence and emotions shape learners’ self-perceptions in the target language. *The Language Learning Journal*, 1-14. <https://doi.org/10.1080/09571736.2024.2447322>

*Reza Valizadeh, M., and Alavinia, P. (2013). Listening comprehension performance viewed in the light of emotional intelligence and foreign language listening anxiety. *English Language Teaching*, *6*(12). <https://doi.org/10.5539/elt.v6n12p11>

*Roohani, A. (2009). The study of emotional intelligence and literature in education: Gender and major of study. *Journal of Asia TEFL*, *6*(4).

*Roohani, A., Etesami, R., and Mirzaei, A. (2020). Exploring Learning Styles and Oral Communication Strategies and their Relationship with Emotional Intelligence of EFL Learners. *Teaching English Language*, *14*(2), 1-27. <https://doi.org/10.22132/tel.2020.115042>

*Saadat, M., and Dastgerdi, M. F. (2014). Correlates of L2 writing ability of Iranian students majoring in English. *Procedia - Social and Behavioral Sciences*, *98*, 1572-1579. <https://doi.org/10.1016/j.sbspro.2014.03.580>

*Santoso, D. R., Affandi, G. R., and Basthomi, Y. (2024). ‘Getting stuck’: A study of Indonesian EFL learners’ self-efficacy, emotional intelligence, and speaking achievement. *Studies in English Language and Education*, *11*(1), 384-402. <https://doi.org/10.24815/siele.v11i1.30969>

*Saud, W. I. (2019). Emotional intelligence and its relationship to academic performance among Saudi EFL undergraduate students. *International Journal of Higher Education*, *8*(6), 222. <https://doi.org/10.5430/ijhe.v8n6p222>

*Shafiee Rad, H., and Hashemian, M. (2022). Role of hedonic and eudaimonic well-being in second language learners’ trait emotional intelligence and emotion regulation. *European Journal of Psychology of Education*, *38*(4), 1619-1641. [https://doi.org/10.1007/s10212‑022‑00663‑4](https://doi.org/10.1007/s10212022006634)

*Shahini, A. (2025). Emotional Dimensions of Feedback: How AI and Human Responses Shape ESL Learning Outcomes. *Ampersand*, 100235. <https://doi.org/10.1016/j.amper.2025.100235>

*Shakarami, A., and Khajehei, H. (2015). How emotional intelligence and language learning strategies interact in an EFL setting. *International Journal of Applied Linguistics and English Literature*, *4*(2). <https://doi.org/10.7575/aiac.ijalel.v.4n.2p.229>

*Sharifi, M., and Ahour, T. (2014). On the Correlation among Iranian EFL Learners’ Emotion Al Intelligence, Self Esteem, and Foreign Language Classroom Anxiety. *Modern Journal of Language Teaching Methods*, *4*(4), 384 - 392.

*Sharifi, M. (2015). On the Correlation between Iranian EFL Learners' Self-Regulation Capacity and their Self Efficacy. *Modern Journal of Language Teaching Methods*, *5*(1), 342.

*Shao, K., Yu, W., and Ji, Z. (2013a). An exploration of Chinese EFL students' emotional intelligence and foreign language anxiety. *The Modern Language Journal*, *97*(4), 917-929. [https://doi.org/10.1111/j.1540‑4781.2013.12042.x](https://doi.org/10.1111/j.15404781.2013.12042.x)

Shao, K., Yu, W., and Ji, Z. (2013b). The relationship between EFL students' emotional intelligence and writing achievement. *Innovation in Language Learning and Teaching*, *7*(2), 107-124. <https://doi.org/10.1080/17501229.2012.725730>

*Shao, Y., Wu, J., Li, Y., Lu, Q., and Wang, Z. (2025). The impact of digital technology use on EFL students’ English academic performance: The mediating roles of emotional intelligence and learning engagement. *BMC psychology*, *13*(1), 638. <https://doi.org/10.1186/s40359-025-02967-8>

*Shiravand, N., and Sarani, A. (2015). An Investigation of the Relationship between Emotional Intelligence and Thinking Styles among Iranian EFL Learners. *Modern Journal of Language Teaching Methods*, *5*(2), 355.

*Shirazi, M. A., and Nadoushani, S. M. M. (2016). Emotional intelligence as the predictor of pragmatic competence: a closer look at Iranian EFL learners’ politeness strategies. *The Reading Matrix: An International Online*, *16*(12).

* Skourdi, S., Rahimi, A., and Bagheri, M. S. (2014). The relationship between emotional intelligence, and vocabulary knowledge among Iranian EFL learners. *Procedia-Social and Behavioral Sciences*, *98*, 1785-1793. <https://doi.org/10.1016/j.sbspro.2014.03.686>

* Soodmand Afshar, H., and Rahimi, M. (2016). Reflective thinking, emotional intelligence, and speaking ability of EFL learners: Is there a relation? *Thinking Skills and Creativity*, *19*, 97-112. <https://doi.org/10.1016/j.tsc.2015.10.005>

*Sucaromana, U. (2004). The relationship between emotional intelligence and achievement in English for Thai students in the lower secondary school. In *Educating: Weaving Research into Practice: Volume 3* (pp. 158-164). Nathan, Qld: Griffith University, School of Cognition, Language and Special Education.

*Tabrizi, A. R., and Esmaeili, L. (2016). The relationship between the emotional intelligence and reading comprehension of Iranian EFL impulsive vs. reflective students. *International Journal of English Linguistics*, *6*(6), 221. <https://doi.org/10.5539/ijel.v6n6p221>

*Taheri, H., Sadighi, F., Bagheri, M. S., Bavali, M., and Khajavi, Y. (2019). EFL learners’ L2 achievement and its relationship with cognitive intelligence, emotional intelligence, learning styles, and language learning strategies. *Cogent Education*, *6*(1). <https://doi.org/10.1080/2331186X.2019.1655882>

* Teoh, G. B., and Liau, A. W. (2021). Emotional intelligence in distance learning: A case study of English as a second language via distance learning. *Journal of Language and Education*, *7*(3), 151-165. <https://doi.org/10.17323/jle.2021.12624>

*Teoh, G. B., and Liau, A. W. (2023). Distance learners’ emotional intelligence and perceptions of their situational barriers in learning english. *Turkish Online Journal of Distance Education*, *24*(2), 120-144. <https://doi.org/10.17718/tojde.1048366>

*Thao, L. T., Thuy, P. T., Thi, N. A., Yen, P. H., Thu, H. T., and Tra, N. H. (2023). Impacts of emotional intelligence on second language acquisition: English-major students’ perspectives. *Sage Open*, *13*(4). <https://doi.org/10.1177/21582440231212065>

*Valizadeh, M. (2016). Iranian EFL Students' Emotional Intelligence and Autonomy in Distance Education. *English Language Teaching*, *9*(10), 22-30. <https://doi.org/10.5539/elt.v9n10p22>

*Vahedi, V. S., and Fatemi, A. H. (2015). The role of emotional intelligence and tolerance of ambiguity in academic Iranian EFL learners' willingness to communicate. *Journal of Language Teaching and Research*, *7*(1), 178. <https://doi.org/10.17507/jltr.0701.20>

* Wang, M., and Wang, Y. (2024). A structural equation modeling approach in examining EFL students’ foreign language enjoyment, trait emotional intelligence, and classroom climate. *Learning and Motivation*, *86*, 101981. <https://doi.org/10.1016/j.lmot.2024.101981>

*Wang, W., Rezaei, Y. M., and Izadpanah, S. (2024). Speaking accuracy and fluency among EFL learners: The role of creative thinking, emotional intelligence, and academic enthusiasm. *Heliyon*, *10*(18), e37620. <https://doi.org/10.1016/j.heliyon.2024.e37620>

*Zadorozhna, I., Datskiv, O., and Levchyk, N. (2018). Development of pre-service foreign languages teachers’ emotional intelligence by means of reflection. *Advanced Education*, *5*(10), 62-68. <https://doi.org/10.20535/2410-8286.144538>

*Zafari, M., and Biria, R. (2014). The relationship between emotional intelligence and language learning strategy use. *Procedia - Social and Behavioral Sciences*, *98*, 1966-1974. <https://doi.org/10.1016/j.sbspro.2014.03.630>

*Zarezadeh, T. (2013). The effect of emotional intelligence in English language learning. *Procedia - Social and Behavioral Sciences*, *84*, 1286-1289. <https://doi.org/10.1016/j.sbspro.2013.06.745>

*Zare‎, J., and Aqajani Delavar‎, K. (2023). The Role of Data-Driven Learning in Developing Trait Emotional‎ Intelligence. *Applied Research on English Language*, *12*(2), 155-174.

*Zhang, C. (2023). The Effects of Emotional Intelligence on Students' Foreign Language Speaking: A Narrative Exploration in China's Universities. *Qualitative Report*, *28*(12). <https://doi.org/10.46743/2160-3715/2023.6296>

*Zhang, X., Davarpanah, N., and Izadpanah, S. (2023). The effect of neurolinguistic programming on academic achievement, emotional intelligence, and critical thinking of EFL learners. *Frontiers in Psychology*, *13*. <https://doi.org/10.3389/fpsyg.2022.888797>

*Zhang, J., and Zhang, W. (2023). Trait emotional intelligence and willingness to communicate in foreign language learning. *Journal of Language Teaching and Research*, *14*(6), 1535-1540. <https://doi.org/10.17507/jltr.1406.11>

*Zhang, T., Zhang, R., and Peng, P. (2024). The relationship between trait emotional intelligence and English language performance among Chinese EFL university students: The mediating roles of boredom and burnout. *Acta Psychologica*, *248*, 104353. <https://doi.org/10.1016/j.actpsy.2024.104353>

*Zhao, J., Li, X., Wei, J., Long, X., and Gao, Z. (2025). Understanding the psychological pathways to translation technology competence: emotional intelligence, self-esteem, and innovation capability among EFL students. *BMC psychology*, *13*(1), 66. <https://doi.org/10.1186/s40359-025-02400-0>

*Zou, X., and Park, H. (2024). The interrelationships of L2 willingness to communicate, emotional intelligence, foreign language classroom anxiety and teacher immediacy. *The Journal of Asia TEFL*, *21*(1), 53-73. <https://doi.org/10.18823/asiatefl.2024.21.1.4.53>
