# Supplementary material for: Emotional intelligence and English as a second/foreign language learning: a systematic review using TCCM framework
Source: Front Psychol. 2026 Jan 9;16:1722555. doi: 10.3389/fpsyg.2025.1722555 (PMC12827750; doi:10.3389/fpsyg.2025.1722555)
Supplement: Supplementary file 2 [file Table_1.docx]

Table 1: Details of theories, context, characteristics and methods of 96 articles

| S.No | Authors (year) | Theory | Context | | Characteristics | | Methodology | | |
| --- | --- | --- | --- | --- | --- | --- | --- | --- | --- |
|  |  |  | **Country** | **Sample Size** | **Independent Variable(s)** | **Dependent Variable(s)** | **Data Collection Method** | **EI Scale** | **Data Analysis Tools** |
|  | Zhao et al. (2025) |  | China | 663 US | EI, SE, IC | TTC | Survey | WLEIS | SEM |
|  | Shao et al. (2025) | SDT, SCgT | China | 931 HSS | DTU, EI and LE (mediator) | ELP | Survey | WLEIS | cor., SMA, CFA |
|  | Li and Pan (2025) | CVT, BBT, SDT, CAST | China | 258 UGUS | EI, TS, (FLE and FLA mediator) | WTC | Survey | TEIQue - SF | cor., and reg. |
|  | Long and Zhu (2025) |  | China | 426 UGS | EI | L2 WFL | Survey | TEIQue - SF | LPA and ANOVA |
|  | Gao et al. (2025) |  | China | 476 US | EI | WTC | Survey | WLEIS | LPA and ANOVA |
|  | Resnik et al. (2025) |  | Austria, Ger. and Swis. | 768 HSS and US | EI | FLA and FLE | Survey | TEIQue - SF | cor. |
|  | Aljasir (2024) |  | Saudi Arabia | 362 Adult learners | EI | Engagement in EFL | Mixed | EILLQ | reg. |
|  | Jin et al. (2024) | CVT and SCT | China | 203 CS | TEI, SE, FLCA | FLP | Survey | TEIQue - SF | cor. |
|  | Namaziandost et al. (2024) | ToM | Iran | 218 US | TEI, SA, CC, AEg and Mindset | FLEd | Mixed | TEIQue - SF | SEM |
|  | Wang and Wang (2024) | BBT and CVT | China | 346CS | EI, CC | FLE | Survey | TEIQue - SF | SEM |
|  | Wang et al. (2024) | SCT | Iran | 320 LIS | EI, CT and AE | AFS | Survey | SSEIT | cor. and SEM |
|  | Zhang et al. (2024) | CVT | China | 489 CS | EI | ELP | Survey | TEIQue - SF | SEM |
|  | Li and Zhang (2024) | SDT; AT and BBT | China | 806 US | TSR (EI- Mediator) | FLE and BO | Survey | WLEIS | cor. |
|  | Zou, and Park (2024) |  | China | 1040 US | EI, WTC, FLCA, TI |  | Survey | WLEIS | SEM |
|  | Santoso et al. (2024) |  | Indonesia | 100 US | EI, SE | SpA | Survey | ERQ | cor. and reg. |
|  | Gao and Yang (2023) | CVT | China | 391 US | EI and ERS | NEI | Survey | TEIQue - SF |  |
|  | Thao et al. (2023) | SEM | Vietnam | 105 US | EI | SLA | Mixed | Self-designed | DS |
|  | McEown et al. (2023) | SDT | Japan | 184 UGS | EI | AS, BO, Aeg | Survey | TEIQue - SF | SEM |
|  | Zhang and Zhang (2023) |  | China | 181 CS | EI | WTC | Survey | TEIQue - SF | cor. and reg. |
|  | Zhang (2023) | TRD | China | 2 CS | EI | S | Qual |  | Thematic Ana. |
|  | Imamyartha et al. (2023) |  | Indonesia | 371 US | EI | LE, Motivation and Achievement | Survey | TEI | SEM |
|  | Farsad and Modarresi (2023) |  | Iran | 126 US | EI | L2 Ego components | Mixed | SSEIT | SEM |
|  | Teoh and Liau (2023) |  | Malaysia | 238 DL | EI | SBLE | Mixed | SSEIT | Thematic Analysis |
|  | Shafiee Rad and Hashemian (2022) |  | Iran | 312 LIS | EI and ER | WB | Survey | TEIQue - SF | cor. and reg. |
|  | Algraini (2022) |  | Saudi Arabia | 100 US | EI | MRS | Survey | TEIQue - SF | cor. |
|  | Han et al. (2022) |  | China | 615 US | FLA (EI – Mediator; CE – Mod.) | Academic Success | Survey | BEIS | cor. and reg. |
|  | Alrabai and Alamer (2022) | BBT | Saudi | 485 UGS | EI, FLA, FLE, boredom and effort | resilience in L2 learning | Survey | TEIQue - SF | SEM |
|  | Resnik and Dewaele (2021) | BBT | Across Europe | 510 CS | LA | FLA and FLE | Survey | TEIQue - SF | cor. |
|  | Chen et al. (2021) | GTP and CDST | China | 274 PGS | EI, FLA, FLE |  | Survey | TEIQue - SF | cor.and reg. |
|  | Bata and Castro (2021) |  | Spain | 6 US | EI | SE | Mixed | TMMS | MAXQDA |
|  | Li et al. (2021) |  | China | 1718 SSS and 1295 US | EI and CE | FLA and FLE | Survey | TEIQue - SF | cor. and reg. |
|  | Dastgoshadeh, & Javanmardi (2021) |  | Iran | 67 US | EI | WTC | Survey | SSEIT | cor., reg., and ANOVA |
|  | Jalilzadeh &Yeganehpour, (2021) |  | Iran | 86 US | EI, WTC | S. performance | Survey | BEQI | cor. and reg. |
|  | Resnik et al. (2021) |  | Across Europe | 481TS | FL Grit (TEI mediator) | FLA and FLE | Mixed | TEIQue - SF | cor. and reg. |
|  | Mede and Budak (2021) |  | Türkiye | 148 US | EI | FLA and DF | Survey | BEQI | cor. |
|  | Teoh and Liau (2021) |  | Malaysia | 238 DL | EI | DLE | Mixed | SSEIT | cor. and reg. |
|  | Aliasin and Abbasi (2020) |  | Iran | 102 LIS | EI | MRS | Survey | BEQI | cor. and reg. |
|  | Chen and Zhang (2020) |  | China | 72 PG US | EI | ELP | Survey | TEIQue – SF | cor.and reg. |
|  | Resnik and Dewaele (2020) |  | 4 European countries | 768 HSS and US | EI | FLA and FLE | Mixed | TEIQue - SF | cor. |
|  | Ożańska-Ponikwia et al. (2020) |  | Poland | 132 UGS | Neuroticism  (EI - Mediator) | S. Performance | Survey | TEIQue - SF | cor. and reg. |
|  | Hamdzah et al. (2020) |  | Malaysia | 250 UGS | EI | MUET performance | Survey | TEQI | cor. and reg. |
|  | Roohani et al. (2020) | ILT | Iran | 108 US | EI | LS and OCP | Survey | BEQI | cor. |
|  | Li (2019) | BBT, WBT, CVT | China | 1307HSS | EI, learning achievement | FLE | Survey | TEI SF | cor. |
|  | Mujiono et al. (2019) | MIT | Indonesia | 100 US | VLI and EI | AA | Survey | SSEIT | cor. and reg. |
|  | Taheri et al. (2019) |  | Iran | 188 US | EI, cognitive intelligence | L2 achievement, LSS | Survey | BEQI | cor. |
|  | Farahani and Gholam-Shahbazi (2019) | ST | Iran | 39 LIS | EI | L. performance in IELTS | Survey | BEQI | cor. |
|  | Saud (2019) |  | Saudi | 80 UGS | EI | EAT | Survey | SSEIT | ANOVA and t- test |
|  | Mohammadi and Izadpanah (2018) |  | Iran | 40 LIS | EI | W. proficeincy | Survey | TEIQue - SF | cor. |
|  | Abdolrezapour (2018) |  | Iran | 102 SS | EI | oral performance | Survey | TEIQue -ASF | cor. |
|  | Mehrpoor and Soleimani (2018) |  | Iran | 340 LIS | EI | WTC, SCCCA | Survey | BEQI | cor. and reg |
|  | Khaghaninejad et al. (2017) |  | Iran | 75 LIS | EI, IQ, self-esteem | R. comprehension | Survey | BEQI | cor. and reg. |
|  | Manzouri and Movahed (2017) |  | Iran | 82 UG US | EI | FLA, SRLP, LAc | Survey | TEI QUE SF | cor. |
|  | Méndez López and Bautista Tun (2017) |  | Mexico | 20 US | EI | MDSA | Qual | TMMS | Thematic analysis |
|  | Khademi and Farokhmehr (2017) |  | Iran | 150 US | EI | SP in e - learning | Survey | BEQI | cor. and reg. |
|  | Hamidrez and Maryam (2016) |  | Iran | 150 US | EI | S. Proficiency in e-learning | Survey | BEQI | cor. and reg. |
|  | Afshar et al. (2016) |  | Iran | 138 US | EI, LS and LLS | L2 achievement | Survey | BEQI | cor. and reg. |
|  | Soodmand Afshar and Rahimi (2016) |  | Iran | 150 US | EI and RT | APS | Survey | BEQI | cor. and reg. |
|  | Shirazi & Nadoushani (2016) |  | Iran | 150 UG/PG US | EI | PC | Survey | EIA | cor. and reg. |
|  | Valizadeh (2016) |  | Iran | 110 DE learners | EI | autonomy | Survey | BEQI | cor. |
|  | Ghaemi and Kodabakhsh (2016) |  | Iran | 34 US | EI | level of RE | Survey | BEQI | cor. and reg. |
|  | Korpi and Farvardin (2016) |  | Iran | 124 US | EI | WCAF | Survey | BEQI | cor. |
|  | Atapour (2016) |  | Iran | 52LIS | EI | learning style | Survey | BEQI | cor. |
|  | Tabrizi and Esmaeili (2016) |  | Iran | 121 HSS | EI | R. comprehension | Survey | BEQI | cor. |
|  | Karimi et al. (2016) |  | Iran | 75 LIS | EI and CT | L2 VK | Survey | BEQI | cor. and reg. |
|  | Mohazabieh and Sadighi (2016) |  | Iran | 30US | EI | VLS | Survey | SSEIT | cor. |
|  | Vahedi and Fatemi (2015) |  | Iran | 64 US | EI and ToA | WTC | Survey | BEQI | cor. |
|  | Shakarami and Khajehei (2015) |  | Iran | 84US | EI | LLS | Survey | BEQI | cor. |
|  | Shiravand and Sarani (2015) |  | Iran | 90 US | EI | Thinking styles | Survey | BEQI | cor. |
|  | Oz (2015) |  | Türkiye | 165 US – TE | EI | WTC | Survey | SSEIT | cor. and reg., ANOVA |
|  | Amini and Sabber (2015) |  | Iran | 150 US | EI | WTC | Survey | BEQI | cor. |
|  | Sharifi (2015) |  | Iran | 100 US | EI | Self - regulation | Survey | BEQI | cor. |
|  | Ghabanchi & Rastegar (2014) |  | Iran | 55 US | EI, IQ | R. comprehension | Survey | BEQI | cor. |
|  | Ghanadi and Ketabi (2014) |  | Iran | 138 US | EI | Belief about LL | Survey | TEIQue - SF | cor. and reg. |
|  | Saadat and Dastgerdi (2014) |  | Iran | 62 US | EI | W. ability | Survey | MMIQ (part) | cor. and reg. |
|  | Alavinia and Alikhani (2014) |  | Iran | 200 US | EI | WTC | Survey | BEQI | cor. and t- test |
|  | Niroomand et al. (2014) |  | Iran | 54 US | EI | Motivation | Survey | SSEIT | cor. |
|  | Rahimi et al. (2014) |  | Iran | 80 PGS | EI | PP | Survey | BEQI | cor. |
|  | Skoudri et al. (2014) |  | Iran | 103 US | EI | VK | Survey | SSEIT | cor. and reg. |
|  | Mahasneh (2014) |  | Jordan | 720 US | EI | Metacognition | Survey | EIQ | cor. and reg. |
|  | Zafari and Biria (2014) |  | Iran | 100 US | EI | usage of LLS | Survey | BEQI | cor., reg.,  ANOVA, MANOVA. |
|  | Sharifi and Ahour (2014) |  | Iran | 200 US | EI and Self Esteem | FLCA | Survey | BEQI | cor. |
|  | Afshar and Rahimi (2014) |  | Iran | LIS | EI and CT | Speaking abilities | Survey | BEQI | cor. and reg. |
|  | Bagheri and Ghasemi (2013) |  | Iran | 30 LIS | EI | W. performance | Survey | SSEIT | cor. and ANOVA |
|  | Zarezadeh (2013) |  | Iran | 330 US | EI | ELL | Survey | BEQI | cor. |
|  | Reza Valizadeh and Alavinia (2013) |  | Iran | 160 US | EI and FLLA | Performance in L. | Survey | BEQI | cor. and reg. |
|  | Shao et al. (2013a) |  | China | 510 US | EI | FLCA | Survey | TEIQue - SF | cor. and reg. |
|  | Karimi (2012) |  | Iran | 92US | EI | W. skills | Survey | SSEIT | cor. |
|  | Alavinia and Behyar (2012) |  | Iran | 100 US | EI | lexico-semantic errors | Survey | BEQI | cor. |
|  | Alavinia & Mollahossein (2012) |  | Iran | 112 US | EI | Metacognitive listening strategies | Survey | BEQI | cor. and reg. |
|  | Alavinia and Ebrahimpour (2012) |  | Iran | 132 US | EI | LS | Survey | BEQI | cor. |
|  | Ożańska-Ponikwia (2012) |  | Ireland and England | 102 bilinguals | Personality Traits and EI | 'Feeling differnet' while using a FL | Survey | TEIQue - SF | cor. and reg. |
|  | Nesari et al. (2011) |  | Iran | 120 LIS | EI | FLVL | Survey | BEQI | cor. |
|  | Ghonsooly and Mazaheri (2010) |  | Iran | 120 US | EI | DSAP | Survey | BEQI | Chi-square |
|  | Roohani (2009) |  | Iran | 345 UG and PGS | EI | IoL | Survey | MSCEIT  EQ Map | cor. and MANOVA |
|  | Dewaele et al. (2008) |  | different countries | 464 adult multilinguals | EI and Socio biographical variables | CA and FLA | Survey | TEIQue - SF | ANOVA and cor. |
|  | Sucaromana (2004) |  | Thailand | 273 SSS | EI | EA | Survey | BEQI | SEM |

Abbreviations used in the Table 1 and 2

| Theories: CVT - Control Value Theory, SCgT - social cognitive theory, SCT - Socio Cultural Theory, ToM - Theory of Mindset, SEM - Socio-educational Model, BBT - Broaden and Build Theory, TRD – Bandura’s triadic reciprocal determinism, SDT - Self-determination Theory, AT- Attachment Theory, GTP - Grand Theory of Personality, CDST - Complex Dynamic Systems Theory, ILT - Interlanguage Theory, WBT - Well-being Theory, MIT - Multiple Intelligences Theory, ST - Schema Theory, CAST - cognitive affective system theory, SET - self - efficacy theory, AT – Attribution Theory, |
| --- |
| Characteristics: TEI - Trait Emotional Intelligence; SE - self - esteem; IC - innovation capability; TTC - translation technology competence; SEf - Self - efficacy; FLCA - Foreign Language Classroom Anxiety; PC – pragmatic competence; FLP - Foreign Language Performance; SA - Self- assessment; LE - language engagement; CC - classroom climate; AEg - Academic Engagement; FLEd - Foreign Language Education; DTU - digital technology usage; TS - teacher support; ERS - Emotion Regulation Strategies of Cognitive reappraisal and expressive suppression; NEI - Negative emotion intensity; SLA - second language acquisition; ER - Emotion Regulation; WB - Well-being; FLE - Foreign language enjoyment; S - Speaking; L - Listening; W - Writing; ELP - English Language Performance; LA - Learner Autonomy; FLA - Foreign Language Anxiety; TSR - Teacher student relationship; BO - Burnout; WTC - Willingness to Communicate; TI - Teacher Immediacy; SpA - Speaking Achievement; AS - Academic Stress; DLE - Distance learning of English; MRS - Metacognitive reading strategies; Speaking Exam; CE - classroom environment; MUET - Malaysian University English Test; LS - learning style; OCP - Oral Communication performance; VLI - Verbal Linguistic Intelligence; AA - Academic Achievement; EAT -English Achievement Test; LL - language learning; LSS – learning style and strategies; FLLA - Foreign Language Listening Anxiety; LLS - Language learning strategy; RT - reflective thinking; APS - ability and proficiency in speaking; ToA - Tolerance of Ambiguity; CA - communicative anxiety; CT - critical thinking; AFS - accuracy and fluency in speaking; AE - Academic Enthusiasm; SRLP - Self - rated Language Proficiency; LAc - Language Achievement; DF - demotivating factors; ELL - English language learning; RE - reading engagement; WCAF - Writing complexity, accuracy and fluency; MDSA - Motivating and demotivating factors in speaking activities; SP - speaking proficiency; FLVL - foreign language vocabulary learning; VK - vocabulary knowledge; VLS - vocabulary learning strategies; DSAP - directive speech act performance; SCCCA - self - perceived communication competence and communication apprehension; IoL - interpretation of literature; SBLE - situational barriers in learning English; PP - Pragmatic Performance; EA - English Achievement; WFL - writing feedback literacy. |
| Population: LI - language institute students, DL - distance learners, US- University students, CS - College Students, UG - Undergraduate students, PG - post graduate students, HSS - High School Students, SSS - secondary school students |
| EI Scales: EILLQ - Emotional Intelligence in Language Learning questionnaire, TEIQue-SF - Trait Emotional Intelligence Questionnaire – Short Form), EIA – Emotional Intelligence Appraisal, WLEIS - Wong and Law Emotional Intelligence Scale, SSEIT - Schutte’s Self-Report Emotional Intelligence Test, ERQ - Emotion Regulation Questionnaire, TMMS - Trait Meta - Mood Scale, BEQI - Bar-On Emotional Quotient Inventory, TEQI - Tapia’s Emotional Quotient Inventory, TEI – Trait Emotional Intelligence (150 items), BEIS – Brief Emotional Intelligence , ECI - Emotional Competence Inventory, TEIQue-ASF - Trait Emotional Intelligence Questionnaire Adolescent SF, SEIQ - Sherer Emotional Intelligence Questionnaire (SEIQ), MMIQ - Mackenzi’s Multiple Intelligences Questionnaire, EIQ - Emotional Intelligence Questionnaire. |
| Statistic Tools: Cor. – correlation, reg., - regression, ANOVA – analysis of variance, ANCOVA – analysis of covariance, MANOVA – multiple analysis of variance, SEM – structural equation modelling, LPA – latent profile analysis, SMA – sequential mediation analysis. |
