# Supplementary material for: Emotional intelligence and English as a second/foreign language learning: a systematic review using TCCM framework
Source: Front Psychol. 2026 Jan 9;16:1722555. doi: 10.3389/fpsyg.2025.1722555 (PMC12827750; doi:10.3389/fpsyg.2025.1722555)
Supplement: Supplementary file 3 [file Table_2.docx]

Table 2: Details of theories, context, characteristics and methods of 25 articles (experimental – based)

| S No | Author(s) | Theory | Country and Samples | Purpose of Enhancing EI | Type of study | Scale to Measure EI | Intervention Method | Statistical Tool |
| --- | --- | --- | --- | --- | --- | --- | --- | --- |
| 1 | Mohammed and Khalid (2025) | SDT, SCT, SET | Iraq  322 US | to verify a method’s effectiveness | mixed method: quasi experimental and semi-structured interview | TEIQue – SF | feedback using chat-gpt to develop writing | ANCOVA |
| 2 | Shahini (2025) | BBT | Canada  63 LIS | to verify a method’s effectiveness | Experimental design | TEIQue – SF | feedback by AI and human teacher for speaking and writing | SEM |
| 3 | Abdolrezapour and Ghanbari (2025) |  | Iran  46 UGS | to verify its effect on writing | concurrent mixed method (experiment and interview) | Not measured | text with emotional content and instruction on emotional literacy | ANOVA |
| 4 | Zare and Aqajani (2023) |  | Iran  92 UG learners | to verify a method’s effectiveness | explanatory sequential design: survey, semi-structured interview, quasi-experimental | TEI Que-SF | Data-driven learning (technology – mediated activities) | Mann-Whitney  U tests |
| 5 | Zhang et al. (2023) |  | Iran  50 female advanced learners | to verify a method’s effectiveness | experimental design | Sherer EI questionnaire 33 items | activities based on Neuro-linguistic Programming (NLP) | ANCOVA |
| 6 | Khaki and Chalak (2022) |  | Iran  50 LIS | to enhance listening performance | quasi-experimental design | TEI Que – ASF | Listening to content with cultural resources | ANOVA, t- test and post-hoc test |
| 7 | Abdolrezapour and Ghanbari (2022) |  | Iran  67 EFL learners | to facilitate perceived flow | mixed method experimental study and semi-structured interview | TEI Que – SF | Activities based on Daniel Goleman’s EI model | ANOVA |
| 8 | David et al. (2021) | SCT | Indonesia  475 US | to verify a method’s effectiveness | quasi-experimental design | 15 items based on Petrides | Mobile learning – chat groups | correlation and t-test |
| 9 | Ghanbari and Abdolrezapour (2021) |  | Iran  67 EFL learners | to verify a method’s effectiveness | quasi-experimental design | TEI Que – SF | EI integrated writing | ANOVA |
| 10 | Alavi and Esmaeilifard (2021) |  | Iran  56 US | to develop L2 achievement and WTC | quasi- experimental | BEQI | Recast feedback through emotional scaffolding | ANCOVA |
| 11 | Andrienko et al. (2020) |  | Ukraine  124 UG students | to verify the effectiveness of a method | mixed method - experimental and semi -structured interview | MSCEIT | By presenting informative news with emotions | No Statistical Tool |
| 12 | Li and Xu (2019) | CVT  WBT BBT | China  study1:  1718 HSS  Study 2: 108 HSS | to verify its impact on FLA and FLE | exploratory sequential design study 1: survey with open ended questions. study 2: experimental study and semi-structured interview | TEI Que – SF | ARGUER model to enhance EI and activities based on positive psychology for diary writing | study 1: t-test  study 2: ANCOVA |
| 13 | Ismaeel and Khaleif (2019) |  | Iraq  30 UGUS | to improve academic achievement | pre-test and post-test design | ECI | Content based approach using Daniel Goleman’s EI model | correlation and e t-test. |
| 14 | (Zadorozhna et al. (2018) |  | Ukraine70 pre-service teachers | to verify the effectiveness of a method | experimental design | Self-Rated EI Scale | Reflecting (end of class) and writing journals | MS Excel |
| 15 | Ebrahimi et al., (2018a) |  | Iran  43 Freshmen | to develop reading skills | quasi-experimental design | Persian version of Bar-On EQ | “50 Activities for Developing EI” by Adele B. Lynn | Kolmogorov-Smirnov test, Mann-Whitney Test |
| 16 | Ebrahimi et al. (2018b) | SCT and reading theory | Iran  43 Freshmen | to develop speaking skills | quasi-experimental design | BEQI | “50 Activities for Developing EI” by Adele B. Lynn | Kolmogorov-Smirnov test, Mann-Whitney Test |
| 17 | Ebrahimi et al. (2018c) |  | Iran  43 US | to enhance writing skill | quasi-experimental design | Persian version of Bar-On EQ | “50 Activities for Developing EI” by Adele B. Lynn | Kolmogorov-Smirnov test, Mann-Whitney Test |
| 18 | Abdolrezapour (2016a) | cognitive load theory | Iran  63 LIS | to improve learners' oral fluency | quasi-experimental design | TEIQue-ASF | Computer mediated EI activities | correlation and   t-test. |
| 19 | Abdolrezapour (2016b) | SCT and reading theory | Iran  50 LIS | to improve reading comprehension | quasi-experimental design | TEIQue-ASF | Emotionalised dynamic assessment based on Goleman's EI framework. | ANOVA |
| 20 | Izadi and Nowrouzi (2016) |  | Iran  42 US | to verify a method’s effectiveness | experimental design | TEIQue – SF | reciprocal teaching in reading comprehension | Paired sample t-test |
| 21 | Shao et al. (2013b) |  | China  68 US | to verify the effectiveness of a method | quasi-experimental design | TEIQue-ASF | Specially designed writing lessons | correlation and   t-test |
| 22 | Barzegar and Sadr (2013) | AT | Iran  136 UG students | to increase motivation | experimental design | Not measured | EI raising activities based on 15 EI skills | t-test |
| 23 | Abdolrezapour et al. (2013) | SCT | Iran  50 LIS | to verify the effectiveness of a method | quasi-experimental design | TEIQue Adolescent SF | Emotionalised dynamic assessment based on Goleman's EI framework. | ANOVA |
| 24 | Abdolrezapour (2013) |  | Iran  44 female LIS | to improve writing performance | experimental method | TEIQue-ASF | Reading comprehension with emotionally loaded content | t-test |
| 25 | Abdolrezapour and Tavakoli (2012) |  | Iran  63 LIS | to enhance reading comprehension | quasi-experimental design | TEIQue-ASF | Reading comprehension with emotionally loaded content | t-test |
